# *Pseudomonas aeruginosa* PfpI is a methylglyoxalase

**DOI:** 10.1016/j.jbc.2025.108374

**Published:** 2025-03-03

**Authors:** Larson Grimm, Andre Wijaya, Isabel Askenasy, Rahan Rudland Nazeer, Hikaru Seki, Paul D. Brear, Wendy Figueroa, David R. Spring, Martin Welch

**Affiliations:** 1 Department of Biochemistry, University of Cambridge, Cambridge, UK; 2 Yusuf Hamied Department of Chemistry, University of Cambridge, Cambridge, UK

**Keywords:** detoxification, enzyme structure, metabolism, microbial metabolism, methylglyoxal, mutant, proteomics, *Pseudomonas aeruginosa*, ThiJ/DJ-1/PfpI superfamily

## Abstract

*Pseudomonas aeruginosa* is an opportunistic pathogen, commonly associated with human airway infections. Based on its amino acid sequence similarity with *Pyrococcus furiosus* protease I, *P. aeruginosa* PfpI was originally annotated as an intracellular protease. In this work, we show that PfpI is a methylglyoxalase. The X-ray crystal structure of the purified protein was solved to 1.4 Å resolution. The structural data indicated that PfpI shares the same constellation of active site residues (including the catalytic Cys112 and His113) as those seen in a well-characterized bacterial methylglyoxalase from *Escherichia coli*, YhbO. Using NMR, we confirmed that PfpI qualitatively converted methylglyoxal into lactic acid. Quantitation of lactate produced by the methylglyoxalase activity of PfpI yielded a *k*_cat_ of 102 min^-1^ and a *K*_*M*_ of 369 μM. Mutation of Cys112 and His113 in PfpI led to complete loss of methylglyoxalase activity. To investigate the functional impact of PfpI *in vivo*, a Δ*pfpI* deletion mutant was made. Quantitative proteomic analyses revealed a pattern of changes consistent with perturbation of ribosomal function, Zn^2+^ limitation, C1 metabolism, and glutathione metabolism. These findings are consistent with PfpI being a glutathione-independent methylglyoxalase. Previously, transposon insertion (*pfpI*::Tn) mutants have been reported to exhibit phenotypes associated with antibiotic resistance, motility, and the response to oxidative stress. However, the Δ*pfpI* mutant generated in this study displayed none of these phenotypes. Whole-genome sequencing of the previously described *pfpI*::Tn mutants revealed that they also contain a variety of other genetic changes that likely account for their observed phenotypes.

*Pseudomonas aeruginosa* is a ubiquitous, Gram-negative, opportunistic pathogen (1, 2, 3, 4). Although *P. aeruginosa* does not typically pose a risk to immunocompetent subjects, it can cause serious infections in immunocompromised individuals or those with underlying health conditions. The airways of people with cystic fibrosis are particularly susceptible to *P. aeruginosa* infections, and indeed, by their “teens,” the airways of many people with cystic fibrosis are permanently colonized by the organism (5). Secreted exoproducts are a major contributor toward the ability of *P. aeruginosa* to infect soft tissues. Thus, *P. aeruginosa* is often considered to be a “professional secretor” and encodes a welter of exceptionally active extracellular proteases whose function is to degrade host tissues yielding nutrients that can support bacterial growth (6, 7, 8). *P. aeruginosa* also encodes several intracellular proteases, many of which are involved in the turnover of cytoplasmic proteins following chemical or environmental insult (9). One protein that was previously included in this category is encoded by *pfpI* (PA0355), so-named because the gene product shares sequence similarity with the *Pyrococcus furiosus* protease I (PfpI; 42% identity over 168 residues, E = 4 × 10^−38^). However, PfpI also shares sequence similarity with the ThiJ/DJ-1/PfpI superfamily (PFAM_PF01965, E = 6 × 10^−45^), which, although largely comprised of peptidases and chaperones, also includes proteins such as DJ-1 (10, 11). The biochemical function(s) of DJ-1 remains unclear, although in humans, the protein has been implicated in cancer, ischemia–reperfusion injury, and autosomal recessive early-onset Parkinson's disease (12, 13, 14). It has been suggested that DJ-1 is a redox-responsive chaperone that prevents α-synuclein aggregation (15, 16, 17), that it binds metal ions, such as Cu^2+^ and Hg^2+^, thereby diminishing their toxicity (18), or that it is a protein–nucleic acid deglycase, helping to repair the damage caused by electrophilic aldehydes in the cell (19).

In the absence of a demonstrable biochemical activity, it is not currently clear whether PfpI functions as a protease or whether it has DJ-1-like activity. To date, most of our understanding of *P. aeruginosa* PfpI function has come from an analysis of PAO1- and PA14-derived mutants containing transposon (Tn) insertions in the gene (20, 21). These studies suggested that PfpI plays a role in the control of motility, biofilm formation, resistance to ciprofloxacin, repair of DNA lesions linked to oxidation, and the response to UV light/heat shock, oxidative stress, ethanol exposure, or osmotic stress (22, 23, 24, 25). However, there are a number of inconsistencies between these studies, likely stemming from differences in the genetic background of the strains studied. For example, two teams independently studied the same PA14 *pfpI*::Tn mutant and reported a -fourfold elevated minimum inhibitory concentration (MIC) for the fluoroquinolone antibiotic, ciprofloxacin (23, 26). By contrast, a third study used a different PA14 *pfpI*::Tn mutant and reported no change in sensitivity to ciprofloxacin (22).

In the current study, and to narrow down the likely function of PfpI in *P. aeruginosa*, we carried out a detailed biochemical characterization of the protein. The X-ray crystal structure of PfpI was similar to that of YhbO, a well-characterized glyoxalase from *Escherichia coli*. This prompted us to further investigate the possible function of PfpI using a combination of biochemical, proteomic, and genetic/genomic approaches. Our data indicate that PfpI is not an intracellular protease but is a methylglyoxalase. Deletion of *pfpI* leads to wholesale changes in the proteome, indicative of a dysregulated stress response. Finally, whole-genome sequencing (WGS) of selected publicly available *pfpI*::Tn mutants revealed that they carry additional genomic changes (aside from the Tn insertion), which likely account for or contribute toward some of the phenotypes previously attributed to *pfpI*. We hypothesize that the primary function of PfpI is in the detoxification of electrophiles such as methylglyoxal (MGO) (22).

## Results

### The crystal structure of PfpI reveals structural similarity with the *E. coli* glyoxalase, YhbO

**Figure 1:**
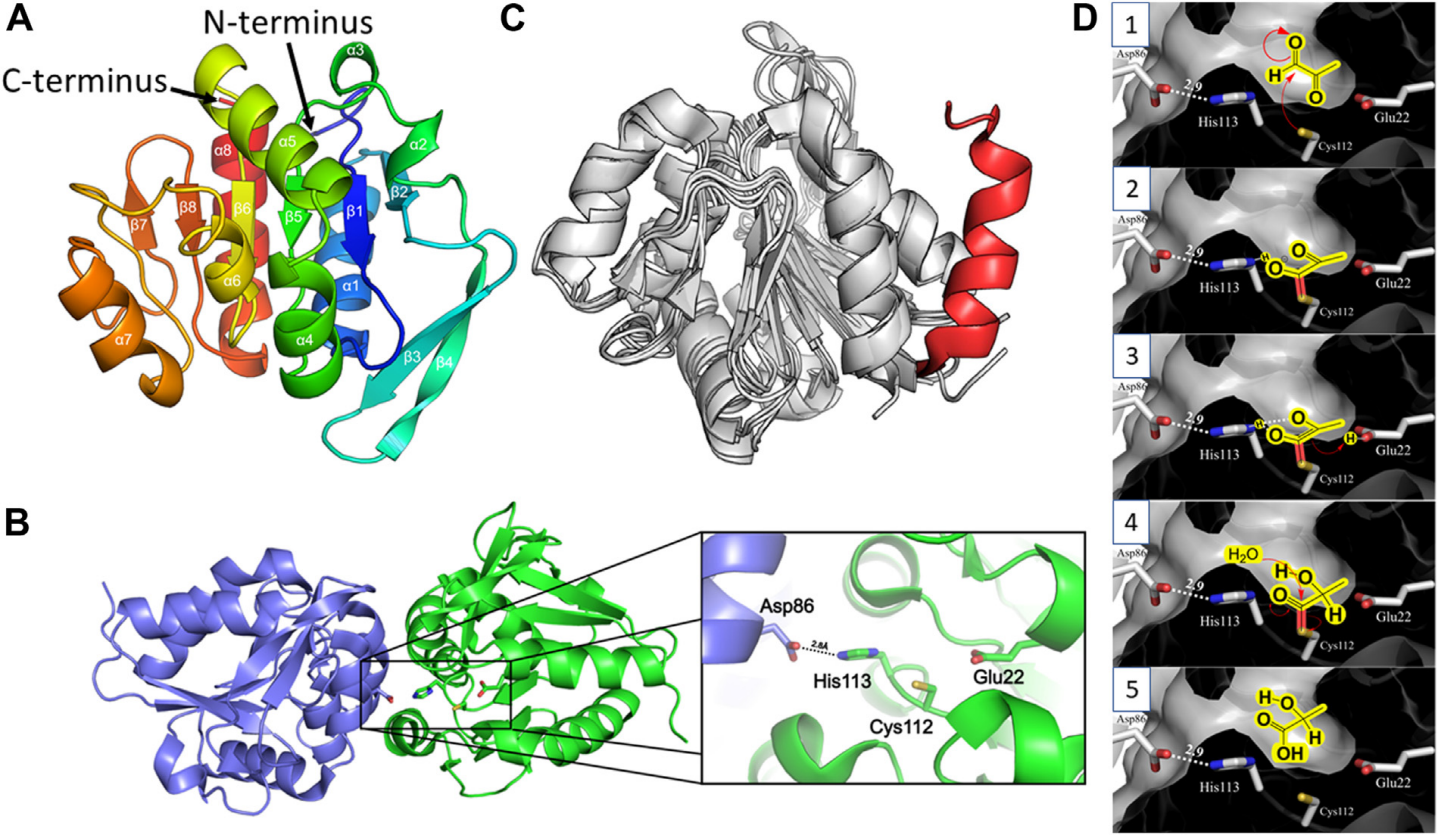
**Overview of the PfpI crystal structure.***A,* chainbow representation of a single *Pseudomonas aeruginosa* PfpI protomer. Each α-helix and β-strand is labeled and depicted in a color gradient (running from the N to the C terminus of the protein). The N and C termini are labeled. *B,* DJ-1 family conserved catalytic residues are located near the dimer interface. The protomers in each PfpI dimer are shown in *green* and *blue*. Key catalytic residues (C112, H113, E22, and D86) are highlighted. C112, H113, and E22 are contributed by one protomer, whereas D86 is contributed by the other. *C,* a *cartoon representation* superposing protomers from *Escherichia coli* YhbO (Protein Data Bank [PDB]: 1OI4), *Pyrococcus horikoshii* PH1704 (PDB: 1G2I), and human DJ-1 (PDB: 5SY6) with a *P. aeruginosa* PfpI protomer. Areas shaded in *gray* share a high degree of structural similarity, with significant overlap of the backbone α-helices and β-strands. However, the α-helix in *red* (amino acids 173–187) belonging to human DJ-1 highlights a major structural difference between these DJ-1 homologs. *D,* proposed catalytic mechanism (1). The nucleophilic side chain of C112 reacts with the aldehyde moiety on the MGO substrate (2). The developing negative charge on the oxygen atom is stabilized by H-bond formation with the side chain of H113 (itself stabilized by H-bond formation with the side chain of D86 from the adjacent protomer) (3). Proton abstraction by the side chain of E22 favors enediol formation (4). Hydrolysis of the thioester bond is accompanied by deprotonation of E22 and release of lactate (5). MGO, methylglyoxal; PfpI, *Pyrococcus furiosus*protease I.

Recombinant PfpI was purified to homogeneity using nickel–nitrilotriacetic acid chromatography. The purified protein behaved as a 38.8 kDa entity in area under the ROC curve analyses (Fig. S1). Given that the predicted molecular mass of monomeric His_6_-tagged PfpI is 21.2 kDa, these data indicate that PfpI is likely to be a globular dimer in solution. Purified PfpI crystallized in the space group P 2_1_ 2_1_ 2_1_ with two molecules in the asymmetric unit. The structure was refined to 1.4 Å resolution with an *R*_cryst_ of 0.254 and an *R*_free_ of 0.274 (Table S3). Each PfpI protomer comprised an α/β sandwich containing eight β-strands and eight α-helices (Fig. 1*A*). As in the other ThiJ/DJ-1/PfpI superfamily members that have been structurally analyzed to date, a β-sheet (comprised of β-strands β1, β2, β5, β6, β7, and β8) forms the core of the structure, which is flanked by helices α1 and α8, and a β-hairpin (incorporating β3 and β4) on one side, and helices α2, α3, α4, α5, α6, and α7 on the other side.

**Figure 2:**
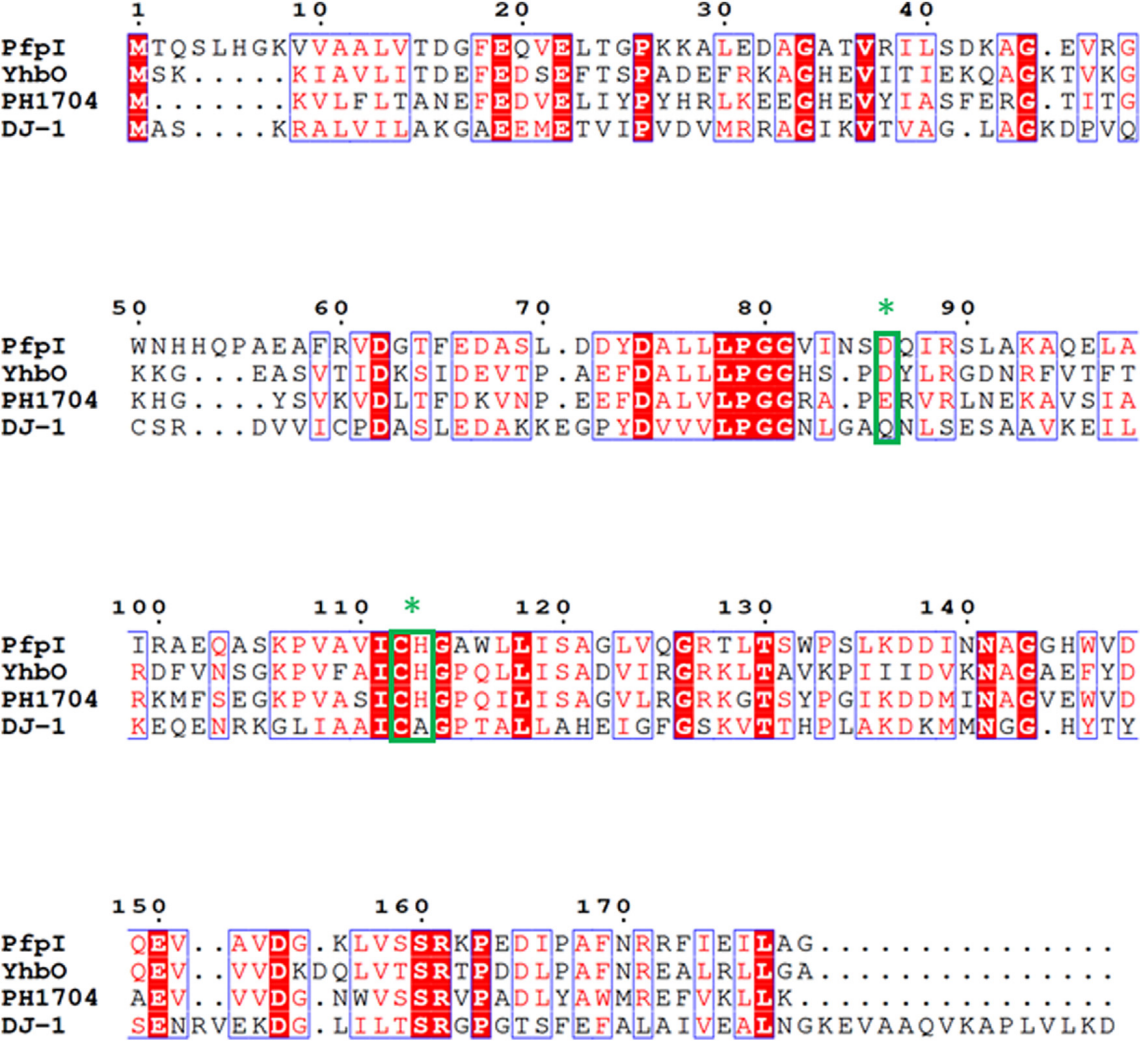
**Amino acid sequence (clustalW) alignment of *Pseudomonas aeruginosa* PfpI with ThiJ/DJ-1/PfpI superfamily members.** PfpI is *P. aeruginosa* PfpI. YhbO is *Escherichiacoli* YhbO (38% sequence identity with PfpI). PH1704 is *Pyrococcus horikoshii* PH1704 (43% sequence identity with PfpI). DJ-1 is *Homo sapiens* DJ-1 (29% sequence identity with PfpI). Numbering is based on PfpI. Regions sharing 100% identity are highlighted in *red*. Regions sharing sequence similarity are boxed in *blue*. The catalytic Cys112, His113, and Asp86 residues are boxed in *green* and highlighted with *asterisks*. PfpI, *Pyrococcus furiosus*protease I.

PfpI and its close structural homolog, PH1704 (a protein with proven proteolytic activity from *Pyrococcus horikoshii* (27, 28), and which shares 43% amino acid identity (E = 5.2 × 10^–38^) with PfpI), both possess a “nucleophile elbow”; a distinctive strand–nucleophile–helix motif and a common characteristic of the “α/β hydrolase” protein fold (29). The α/β fold enzyme family is a large and ever-growing group of structurally related proteins that have diverse catalytic and noncatalytic functions (30, 31). In PfpI, the nucleophile elbow is comprised of residues 108-118 (Fig. 2). The nucleophile itself, Cys112, forms part of a constellation of putative catalytic residues, which includes His113, Glu22, and Asp86 from the adjacent protomer (Fig. 1*B*). In glutathione-dependent glyoxalases, the active nucleophile is also a thiol but contributed by glutathione itself. The residues flanking Cys112 (Val-Ile-Cys-His-Gly-Ala) are broadly commensurate with the consensus sequence (small-x-Nuc-x-small-small) conserved in all α/β hydrolases. PfpI was structurally similar to human DJ-1 and other bacterial DJ-1 homologs (Fig. 1*C*). However, human DJ-1 contains one major difference not seen in the other superfamily members: an additional α-helix (amino acids 173–187) at the C terminus, which contributes to the dimer interface (Fig. 1*C*).

### PfpI has methylglyoxalase activity

**Figure 3:**
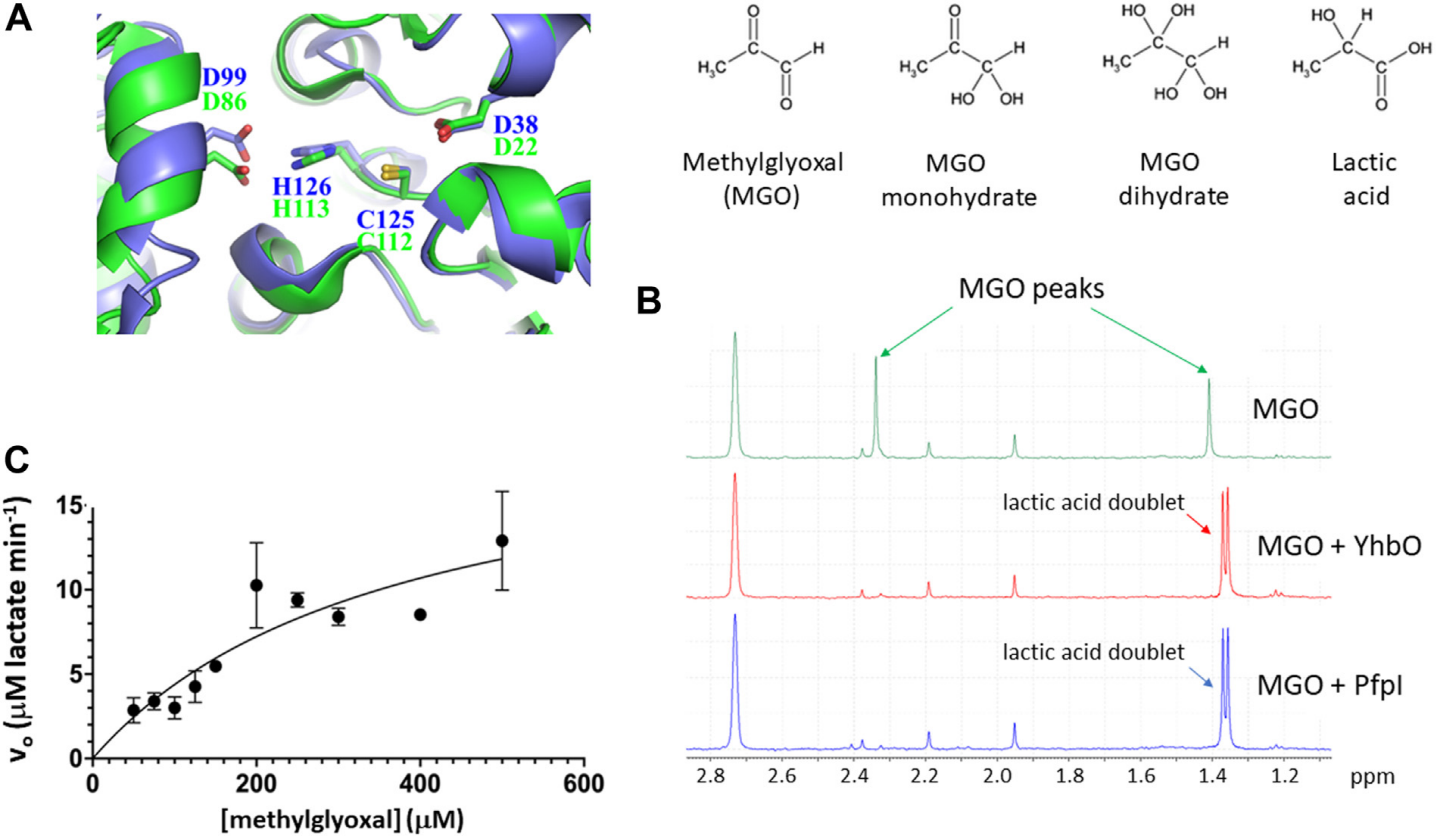
**PfpI has methylglyoxalase activity.***A,* superposition of catalytic residues in PfpI from *Pseudomonas aeruginosa* with the corresponding residues in YhbO from *Escherichia coli*. YhbO (Protein Data Bank code: 1OI4) also crystallized as a dimer, with the catalytic triad located in the cleft between protomers. The PfpI is colored *green*, whereas the YhbO is colored *blue*. *B,* PfpI and YhbO both have methylglyoxalase activity. Purified PfpI (20 μg) or YhbO (20 μg) as indicated, was mixed with MGO (1 mM) and incubated overnight at four ˚C before 1D [^1^H] NMR analysis of the reaction end product(s). Structures of the reactants and products are indicated above the spectra. The *green spectrum* shows the [^1^H] NMR spectrum of MGO incubated overnight without PfpI or YhbO. Diagnostic MGO-associated peaks appear at 1.41 and 2.34 ppm, corresponding to the monohydrated and dihydrated forms of the molecule. These peaks disappear when either YhbO (*red spectrum*) or PfpI (*blue spectrum*) are present, and transition to a doublet at 1.35-1.39 ppm, indicative of methyl group protons coupled to the adjacent methine proton of lactic acid. These data indicate that both enzymes quantitatively convert MGO to lactate. *C,* kinetics of PfpI-catalyzed MGO conversion to lactate. The panel shows how the initial rate of the reaction (*v*_0_) varies with MGO concentration. The mean ± SD for N = 3 independent replicates are shown. Kinetic constants were extracted by fitting a Michaelis–Menten curve to the data using GraphPad Prism. MGO, methylglyoxal; PfpI, *Pyrococcus furiosus*protease I.

MGO is a highly reactive dicarbonyl produced as a byproduct of normal metabolism in many organisms (32). This electrophile needs to be removed to prevent it from chemically modifying DNA, proteins, and other macromolecules. This detoxification is mediated, in part, by glutathione-dependent glyoxalases. However, several DJ-1 family members have been reported to exhibit glutathione-independent glyoxalase activity, including Glx3p from *Candida albicans*, SAV0551 from *Staphylococcus aureus*, and YhbO and HchA (Hsp31) from *E. coli* (33, 34, 35, 36). The structure of YhbO has been solved (Protein Data Bank [PDB] code: 1OI4) and displays the same configuration of active site residues as PfpI (Fig. 3*A*). We therefore hypothesized that PfpI may also display methylglyoxalase activity. Using [^1^H] NMR to monitor the reaction, we found that purified PfpI mediated the same quantitative conversion of MGO to lactate as did purified YhbO (Fig. 3*B*). Note that at neutral pH in aqueous solution, the MGO resonances derive almost exclusively from the monohydrated and dihydrated forms of MGO (*i.e.*, the dicarbonyl form of MGO is not observed) with singlet resonances at 1.378 and 2.306 ppm, as previously reported (37). The other C-*H* proton of MGO appears at 5.287 and 4.806 ppm for the monohydrate and dihydrate forms, respectively. This is beyond the window illustrated in Figure 3*B*. No conversion of the substrate was seen in the absence of PfpI or YhbO. The expected lactate quadruplet (from the methyl group) is seen in the extended [^1^H] spectrum (Fig. S2*A*), and COSY, heteronuclear single quantum coherence, and heteronuclear multiple bond correlation analyses unambiguously confirm the reaction product to be lactate (Fig. S2, *C–E*). Furthermore, spiking the reaction end products with purified lactate yielded no new peaks (Fig. S3). We conclude that PfpI, like YhbO, converts MGO to lactate.

To establish the enzymatic parameters of PfpI, we measured the rate of conversion of MGO to lactate. Reactions were initiated by adding a fixed amount of PfpI to samples containing varying concentrations of MGO. The samples were quenched at differing times by immersion in boiling water. Then, after cooling of the quenched reaction mixtures on ice, the presence of lactate was measured using a recently developed colorimetric procedure (38), yielding initial rates. The initial rates were plotted as a function of MGO concentration to obtain kinetic parameters. These data revealed that PfpI has a *k*_cat_ for MGO of 103 ± 22 min^−1^ and a *K*_M_ of 369 ± 139 μM. There was no production of lactate in assays containing MGO but lacking PfpI or YhbO, indicating that the boiling step used to quench the reactions does not lead to nonenzymatic conversion of MGO to lactate (*data not shown*). We also measured the activity of three active site mutants (C112A, H113A, and H113E) made by site-directed mutagenesis. None of the mutant proteins showed any activity, even after 1-h incubation with 200 μM MGO (*data not shown*). We conclude that Cys112 and His113 are essential for PfpI methylglyoxalase activity. A putative catalytic mechanism involving these side chains and that of an adjacent conserved residue, E22 (Fig. 2), is proposed in Figure 1*D*.

### Impact of PfpI on the *P. aeruginosa* proteome

**Figure 4:**
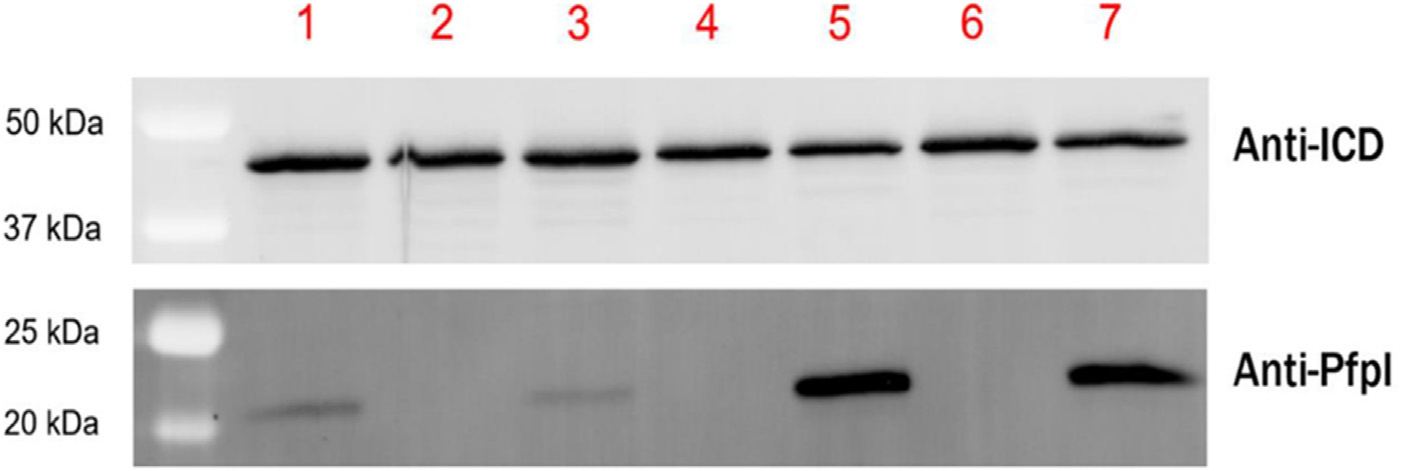
**PfpI expression in *Pseudomonas aeruginosa* PAO1 derivatives.** Cell lysates from the indicated PAO1 derivatives were probed for expression of a loading control (isocitrate dehydrogenase [ICD, 45.6 kDa, *upper panel*]) and for expression of PfpI (19.2 kDa, *lower panel*). Lane 1 = PAO1. Lane 2 = PAO1 Δ*pfpI*. Lane 3 = PAO1 Δ*pfpI* pUCP20(*pfpI*). Lane 4 = PW1654. Lane 5 = PW1654 pUCP20(*pfpI*). Lane 6 = PW1655. Lane 7 = PW1655 pUCP20(*pfpI*). PfpI, *Pyrococcus furiosus* protease I.

To understand better how PfpI might affect cell function, we first generated an in-frame deletion, which removed all but the first 10 codons of the *pfpI* ORF in PAO1. WGS of the Δ*pfpI* mutant revealed that it contained no SNPs or indels (beyond the *pfpI* deletion itself) compared with the progenitor strain. PfpI expression was measured by Western blotting of cell extracts from the Δ*pfpI* mutant and from the PAO1 progenitor strain. We also investigated PfpI expression in two widely used PAO1-derived *pfpI*::Tn mutants from the UWGC library, PW1654 and PW1655 (20). No PfpI expression was detected in the Δ*pfpI* or *pfpI*::Tn mutants (Fig. 4, *lane 3*). PfpI expression was restored to approximately wildtype levels in the Δ*pfpI* mutant by complementation with pUCP20(*pfpI*). Interestingly, complementation of the two *pfpI*::Tn mutants with pUCP20(*pfpI*) led to much higher levels of PfpI expression, suggesting that the genetic background of these mutants supports overexpression of *pfpI* from the plasmid (Fig. 4, *lanes 5* and *7*). Expression of a housekeeping protein, isocitrate dehydrogenase, was essentially the same in all the samples.

**Figure 5:**
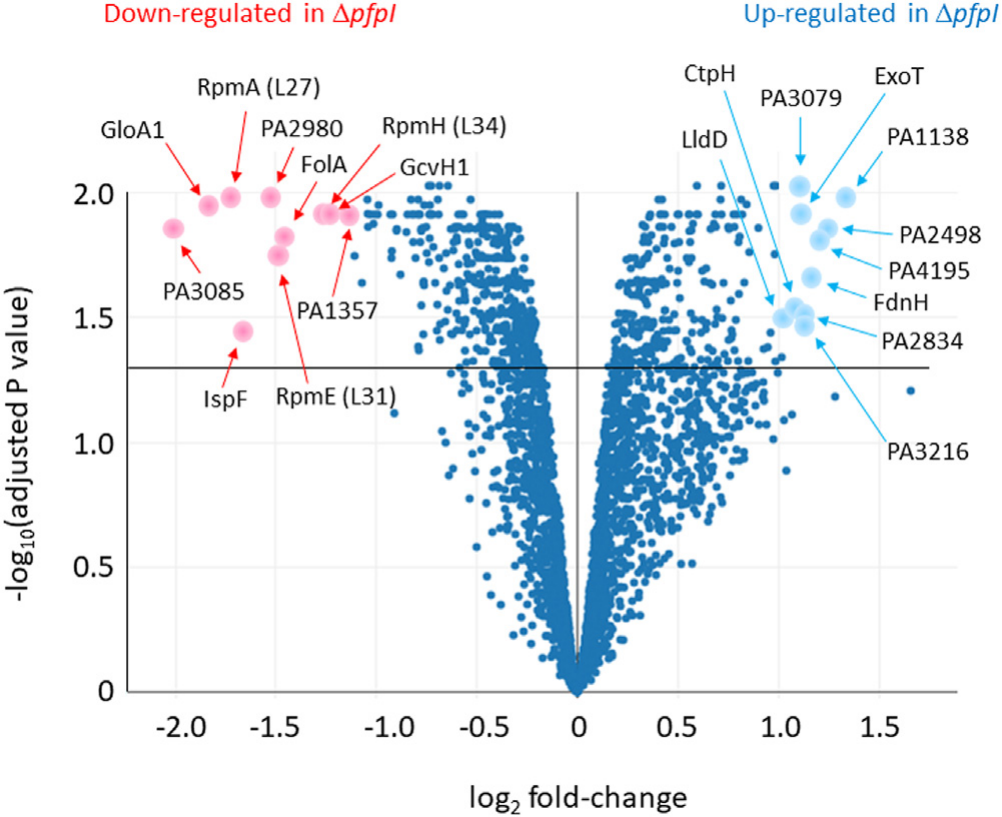
**Protein abundance in the Δ*pfpI* mutant *cf.* the PAO1 progenitor strain assessed using quantitative TMT-based proteomics.** The figure shows a volcano plot of the mean protein abundance in the Δ*pfpI* mutant compared with the PAO1 progenitor strain. The top 10 most highly upregulated and downregulated proteins (*p* < 0.05) in the Δ*pfpI* mutant are highlighted. TMT, tandem mass tag.

We next examined the cell-associated proteome in N = 4 cultures each of the PAO1 progenitor and the Δ*pfpI* mutant using quantitative tandem mass tag–based proteomics. Cultures were grown in phosphate-containing Mops–glucose minimal medium. The goal was to establish which domains of physiology might be affected by the presence (or absence) of PfpI. Peptides from a total of 2878 proteins were quantified. Of these, 18 proteins were found to be significantly (*p* < 0.05) downregulated twofold or more in the Δ*pfpI* mutant, and 11 proteins were found to be significantly (*p* < 0.05) upregulated by more than twofold. A list of all proteins detected, their fold modulation, and statistical significance, in this proteomic study can be found in the Supporting information. The 20 most highly modulated (*p* < 0.05) proteins are listed in Table 1 (proteins with decreased abundance) and Table 2 (proteins with increased abundance), and a “volcano plot” (Fig. 5) highlighting these proteins is shown in Figure 5.

**Table 1:** The 20 most highly modulated proteins (*p* < 0.05) showing decreased abundance in the *pfpI* deletion mutant

	Protein	Locus tag	Description of gene product	Log_2_ FC	*p*
1		PA3085	DUF1315 family protein	−2.009	0.014
2	GloA1	PA3524	Lactoylglutathione lyase	−1.827	0.011
3	RpmA	PA4567	50S ribosomal protein L27	−1.731	0.011
4	IspF	PA3627	2-C-methyl-D-erythritol 2,4-cyclodiphosphate synthase	−1.671	0.036
5		PA2980	UPF0434 protein	−1.529	0.011
6	RpmE	PA5049	50S ribosomal protein L31	−1.475	0.018
7	FolA	PA0350	Dihydrofolate reductase	−1.448	0.015
8	RpmH	PA5570	50S ribosomal protein L34	−1.254	0.012
9	GcvH1	PA5214	Glycine cleavage system protein H1	−1.239	0.012
10		PA1357	YbaK/aminoacyl-tRNA synthetase–associated domain–containing protein	−1.127	0.012
11		PA3566	Putative antibiotic biosynthesis monooxygenase domain–containing protein	−1.109	0.018
12	TesA	PA2856	Esterase	−1.072	0.023
13		PA3331	Cytochrome P450	−1.045	0.011
14	RpsP	PA3745	30S ribosomal protein S16	−1.043	0.012
15		PA3808	Uncharacterized protein	−1.042	0.014
16		PA1890	Probable glutathione-*S*-transferase	−1.031	0.012
17		PA2309	ABC transporter substrate-binding protein	−1.015	0.013
18		PA1033	Probable glutathione-*S*-transferase	−1.012	0.012
19	HisH1	PA5142	Imidazole glycerol phosphate synthase subunit	−0.994	0.012
20	NfxB	PA4600	HTH-type transcriptional regulator	−0.989	0.011

**Table 2:** The 20 most highly modulated proteins (*p* < 0.05) showing increased abundance in the *pfpI* deletion mutant

	Protein	Locus tag	Description of gene product	Log_2_ FC	*p*
1		PA1138	Probable transcriptional regulator	1.343	0.011
2		PA2498	Uncharacterized protein	1.248	0.014
3		PA4915	Probable chemotaxis transducer	1.209	0.016
4	FdnH	PA4811	Formate dehydrogenase iron–sulfur subunit	1.174	0.022
5		PA3216	Uncharacterized protein	1.135	0.035
6		PA2834	Probable transcriptional regulator	1.120	0.031
7	ExoT	PA0044	Exoenzyme T	1.118	0.012
8		PA3079	SSD domain–containing protein	1.111	0.009
9	CtpH	PA2561	Methyl-accepting chemotaxis protein	1.079	0.030
10	LldD	PA4771	l-lactate dehydrogenase	1.039	0.032
11		PA5250	TerC family protein	1.016	0.046
12	MexX	PA2019	Resistance-Nodulation-Cell Division (RND) multidrug efflux membrane fusion protein	0.988	0.009
13		PA5257	HemY N-terminal domain–containing protein	0.985	0.047
14	OlsA	PA4351	Lyso-ornithine lipid O-acyltransferase	0.982	0.018
15		PA1877	Probable secretion protein	0.976	0.009
16	AtpE	PA5559	ATP synthase subunit c	0.961	0.036
17		PA2604	Uncharacterized protein	0.947	0.042
18	FliO	PA1445	Flagellar protein	0.927	0.042
19	OpmB	PA2525	Resistance-Nodulation-Cell Division (RND) multidrug efflux outer membrane subunit	0.901	0.014
20	PilW	PA4552	Type 4 fimbrial biogenesis protein	0.856	0.017

Among the proteins displaying lower abundance in the Δ*pfpI* mutant, three were associated with the 50S ribosomal subunit (L27, L31, and L34). L27 and L31 are components of the 50S “central protuberance” (CP). Another CP protein, L33, was also modulated nearly twofold but with slightly lower significance (*p* = 0.08). The CP appears to be an independent structural and functional unit of the ribosome and has been proposed to be a “central coordinator” of peptidyltransferase and tRNA exit site function (39). These data suggest that translational efficacy may be affected in the Δ*pfpI* mutant. L31 and the ribosomal protein S16 (which was also significantly less abundant in the Δ*pfpI* mutant) both require Zn^2+^ for activity (40), suggestive of possible limitation of this metal ion. Consistent with this, two other Zn^2+^-dependent enzymes displayed lower abundance in the Δ*pfpI* mutant (GcvH1 and FolA) (Table 1). Both of these are involved in folate-dependent C1 metabolism. Perhaps the most striking observation from the proteomics was that the glutathione-dependent glyoxalase, GloA1, was nearly fourfold *less* abundant in the Δ*pfpI* mutant. This was surprising, since we anticipated that the absence of PfpI might be compensated by upregulation of alternative aldehyde detoxification enzymes. However, we noted that two glutathione-*S*-transferases (PA1033 and PA1890) were also significantly less abundant in the Δ*pfpI* mutant (Table 1) suggesting that glutathione may be limiting in the Δ*pfpI* mutant. Finally, we noted that NfxB, a repressor of the MexCD-OprJ efflux pump, was less abundant in the Δ*pfpI* mutant. Ciprofloxacin is a known substrate of MexCD-OprJ (41), so the lower abundance of NfxB in the Δ*pfpI* mutant could potentially account for the previously reported elevated ciprofloxacin resistance of *pfpI* mutants (23). However, when we tested the MIC of ciprofloxacin for the Δ*pfpI* mutant, the MIC was identical to that of the parent strain (PAO1) and also to that of a UWGC-derived Tn mutant, PW1654 (Fig. S4). Interestingly, another UWGC mutant, PW1655, displayed slightly greater resistance to ciprofloxacin (Fig. S4). This greater resistance was not reversed by expression of *pfpI* in *trans* from a plasmid (pUCP20). This suggested that the difference in ciprofloxacin sensitivity between PW1654 and PW1655 might be related to non–*pfpI*-associated genomic differences.

It was less straightforward to discern potential physiological patterns in the proteins that were more abundant in the Δ*pfpI* mutant (Table 2). Several membrane-associated proteins, including two methyl chemotaxis proteins and the RND-family efflux pump components, MexX and OpmB, were more abundant as were two uncharacterized LysR-type transcriptional regulators. MexX forms part of an efflux pump (MexXY-OpmD) that exports aminoglycoside antibiotics (42), whereas OpmB is the outer membrane component of the tetrapartite MuxABC-OpmB efflux pump (43). The latter is poorly characterized but does appear to confer resistance against aztreonam, macrolides, tetracycline, and novobiocin (43).

Taken together, our data indicate that loss of PfpI leads to discrete effects on the cell consistent with a role in translational efficacy, the response to Zn^2+^ limitation, and control of glutathione availability.

### PfpI has no detectable protein interaction partners

One possibility that might explain the lower abundance of GloA1 (and possibly, other proteins too) is that PfpI forms a physical complex with these proteins, and that in the absence of such an interaction, the binding partners are unstable. To test this, we replaced the chromosomal copy of *pfpI* with a version that encodes a twin-*Strep* tag for pull-down analyses. We confirmed that the tagged protein was expressed at the same levels as the wildtype (Fig. S5*A*). However, we obtained no evidence that PfpI specifically coimmunoprecipitates with other proteins in pull-down analyses, except for those proteins that show an intrinsic affinity for the beads (Fig. S5*B*). We conclude that PfpI likely does not interact strongly with other proteins in the cell.

### WGS and phenotypic comparison of PAO1-derived Tn mutants with the Δ*pfpI* mutant

One of the widely used UWGC *pfpI* mutants, PW1654, has been previously reported to display altered H_2_O_2_ sensitivity (22) and mutants from PA14 have been shown to display altered swarming motility, ciprofloxacin resistance (23). We confirmed that PW1654 displayed aberrant (*cf.* the PAO1 progenitor) swarming motility and response to H_2_O_2_ (Figs. S6 and S7, respectively) and that a second UWGC *pfpI*::Tn mutant, PW1655, displays aberrant ciprofloxacin resistance, swarming, and response to H_2_O_2_ (Figs. S4, S6 and S7). However, the Δ*pfpI* mutant made in this study displayed none of these phenotypes. Moreover, the ciprofloxacin resistance phenotype of PW1655 and the swarming phenotype of PW1654 and PW1655 could not be restored by complementation with plasmid-borne *pfpI* (Figs. S4 and S6, respectively). These inconsistencies prompted us to investigate whether PW1654 and PW1655 might contain additional mutations (beyond the Tn insertion in *pfpI*) that potentially contribute toward their previously reported phenotypes. This was done using WGS. PW1654 carries a forward-facing ISl*acZ*/tet^R^/hah insertion at position 166/540 in *pfpI*, whereas PW1655 carries a reverse-facing IS*phoA*/tet^R^/hah insertion at position 440/540. These insertions and their locations were confirmed by WGS.

Away from the *pfpI* locus, PW1654 contained just one SNP (relative to the progenitor PAO1), in *mexQ* (PA3522); a synonymous G→A transition at position 2094/3162. Similarly, PW1655 also contained just one SNP, a G→A transition in *nalC* (PA3721) at position 35/642. This gave rise to a Gly→Asp substitution at position 12 in the encoded protein. NalC is an indirect repressor of *mexAB*-*oprM* expression. MexAB-OprN exports, among other compounds, fluoroquinolones, such as ciprofloxacin. Given that the Gly→Asp mutation affects the amino acid sequence at the start of the DNA-binding helix–turn–helix motif in NalC, it seems reasonable to assert that the elevated ciprofloxacin resistance of PW1655 might be attributable to this amino acid substitution.

In addition to the two SNPs just discussed, PW1654 and PW1655 also contained two overlapping, but nonidentical, large deletions spanning 138 ORFs and 125 ORFs, respectively (Table S4). These deletions removed structural and regulatory genes involved in siderophore biosynthesis/uptake (*pvd* and *fpv* genes), glycine/serine metabolism (*gcv* genes), disulfide bond formation (*dsb* genes), drug efflux (*mex* genes), and catechol, toluene, and anthranilate metabolism (*cat*, *xyl*, and *ant* genes), among others. Presumably, the absence of these genes from PW1654 and PW1655 has a significant functional impact, *cf.* the progenitor strain, PAO1. We conclude that PW1654 and PW1655 are not isogenic with their progenitor (PAO1) and that their phenotypes may not solely be attributable to the Tn insertion in *pfpI*.

It is worth noting that although the deletion in PW1654 substantially overlapped with that in PW1655, it was not identical. For example, the response regulator encoded by *czcR* is deleted in PW1654. Loss of *czcR* has been reported to diminish production of the quorum-sensing signals, OdDHL and BHL, and a *czcRS* deletion mutant was reported to display reduced virulence in a *Caenorhabditis elegans* killing model (44). Also, the ORF encoding one of the most highly downregulated proteins in the Δ*pfpI* mutant, OpmB (Table 2), was deleted in PW1654, as was the associated *muxABC* cluster. The deletion in PW1654 also removed the thioredoxin-dependent thiol peroxidase–encoding gene, *tpx*. Tpx plays a role in reversing the thiol oxidation associated with submillimolar concentrations of H_2_O_2_ but does not enhance survival at higher concentrations of the oxidant (45), making it unlikely that the *tpx* deletion in PW1654 contributes toward the response of this strain to H_2_O_2_ challenge (Fig. S7). At this juncture, it is worth noting that the response to H_2_O_2_ challenge in both PW1654 and PW1655 was apparently “complemented” by expression of *pfpI* in *trans* in these strains. Given that this H_2_O_2_ sensitivity was not observed in the Δ*pfpI* mutant (which, relative to the progenitor, verifiably contains *only* the deletion in *pfpI* and no other mutations), we assume that this result is associated with the considerable overexpression of *pfpI* from pUCP20 in the PW1654 and PW1655 backgrounds (Fig. 4). Presumably, this overexpression phenotype is somehow related to the large shared deletion in these strains.

Taken together, we conclude that these genomic differences (along with the *nalC* mutation in PW1655) contribute toward the slightly different phenotypic behaviors of PW1654 and PW1655.

## Discussion

PfpI is a ThiJ/DJ-1 family protein from *P. aeruginosa* that was previously incorrectly annotated as a protease. In this report, we show that PfpI is a methylglyoxalase. The structure of PfpI was similar to that of other DJ-1 superfamily members, and the proposed catalytic residues, Cys112 and His113, were experimentally confirmed to be essential for methylglyoxalase activity. MGO is a toxic α,β-dicarbonyl that can react with amino groups (the Maillard reaction) and thiol groups on enzymes as well as with DNA (principally guanosine bases to yield *N*^2^-(1-carboxyethyl)-deoxyguanosine). These reactions not only directly inactivate biomolecules; in the presence of trace metals, such as Cu^2+^, MGO can also react with lysine leading to the production of DNA-damaging free radicals (46). Therefore, and to moderate MGO accumulation in the cell, *P. aeruginosa* encodes several GSH-dependent MGO detoxification pathways. PfpI now adds a GSH-independent pathway to this cadre.

In many organisms, MGO is produced by methylglyoxal synthase from the glycolytic intermediate, dihydroxyacetone phosphate (DHAP). However, *P. aeruginosa* does not encode a homolog of methylglyoxal synthase, and, given that *P. aeruginosa* exclusively employs the Entner–Doudoroff pathway for glycolysis, it likely does not generate significant quantities of DHAP and thence, MGO. Nevertheless, MGO can be generated, albeit in smaller quantities, from spontaneous fragmentation of triose phosphates derived from, for example, the GlpD-catalyzed conversion of glycerol 3-phosphate to DHAP (47). There are also indications that MGO may be generated downstream as a direct pathway intermediate in *Pseudomonas* spp. during the conversion of glyceraldehyde 3-phosphate to pyruvate under some conditions (48). In addition to these intracellular sources of MGO, there is also evidence to suggest that neutrophils produce the compound as an antibacterial strategy (49). Irrespective of its source, the detoxification of MGO is likely to be more broadly relevant in disease scenarios. For example, toxic electrophiles such as MGO induce expression of a multidrug efflux pump, MexEF-OprN, making infections more difficult to treat (50). Furthermore, *lasR* mutants of *P. aeruginosa* are commonly encountered in the cystic fibrosis airways. LasR is a regulator that sits at the top of the quorum sensing hierarchy in the organism (51, 52, 53), and it was recently shown that *lasR* mutants have lower intracellular GSH levels and concomitantly increased sensitivity to MGO (54). Maintenance of a GSH-independent pathway for detoxifying MGO may therefore be particularly important in these circumstances.

Until recently, MGO detoxification was thought to be mainly mediated by a collection of GSH-dependent GlxI-type glyoxalases. Here, GSH reacts nonenzymatically with MGO to yield an MG–GSH hemithioacetal, which is then converted by GlxI into an *S*-D-lactoylglutathione derivative for subsequent conversion to lactate by a GlxII-type enzyme, GloB. To our knowledge, the GloB enzyme in *P. aeruginosa* has not yet been identified, although based on amino acid sequence similarity, PA1813 likely serves this purpose. *P. aeruginosa* encodes three GlxI-type glyoxalases, GloA1, GloA2, and GloA3, each of which can catalyze the proton transfer and enolization steps that form the lactoylglutathione intermediate. GloA1 and GloA2 are Ni^2+^/Co^2+^ activated, whereas GloA3 is Zn^2+^ dependent. Given that the Δ*pfpI* mutant displays a proteomic signature indicative of being Zn^2+^ limited, *a priori*, we would therefore expect that GloA3 should be less abundant (although we recognize that this inappropriately conflates activity with protein expression level). However, we obtained no evidence for GloA2 or GloA3 expression (see Supporting information for proteomic dataset), and contrary to expectation, GloA1 was among the most highly downregulated proteins in the Δ*pfpI* mutant. It remains unclear why the cell would downregulate its only expressed GSH-dependent glyoxalase, GloA1, and concomitantly downregulate GSH synthesis in the absence of the GSH-independent glyoxalase, PfpI.

The Δ*pfpI* mutant also displayed other significant proteomic modulations. The clearest of these involved ribosomal proteins, some of which showed decreased abundance in the mutant. We speculate that without an efficient system for detoxifying MGO, protein glycation increases and that this is offset by a decrease in the rate of protein synthesis. Our data suggest that this decrease in translation may involve proteins associated with the CP of the ribosome.

The *K*_M_ and *k*_cat_ values of PfpI for MGO were 369 ± 139 μM and 103 ± 22 min^−1^, respectively. The value of *k*_cat_/*K*_M_ for PfpI is therefore ca. 4.7 × 10^3^ M^−1^ s^−1^. By comparison, the *k*_cat_/*K*_M_ value of GloA1 (the only other GlxI-type isozyme expressed in the growth conditions used in this study) for MGO–GSH hemithioacetal substrate is 8.5 × 10^6^ M^−1^ s^−1^ (with Ni^2+^ as a cofactor) (55). Clearly, the GloA1 is far more efficient as a detoxifying enzyme than PfpI. Nevertheless, when GSH is limiting, the cell may have few other options for handling MGO. Also, we note that the previously reported *k*_cat_ value for the *E. coli* DJ-1 family member, YhbO, with MGO as a substrate is 17.4 min^−1^ (at 22 °C) (56), which is considerably slower than the turnover by PfpI reported here. It therefore seems likely that the DJ-1 family of methylglyoxalases are generally less efficient than their GlxI family counterparts. Another formal possibility that is worth considering is that although PfpI demonstrably catalyzes the conversion of MGO to lactate, it might also act on other substrates, for example, glycated proteins or glyoxylate. To test the former, we tried including glycated bovine serum albumin into the reaction mixtures, but unfortunately, this interfered with the lactate detection assay, so we were unable to quantify the kinetics of this. However, the latter is certainly true, since we observed quantitative conversion of glyoxal to glycolate by PfpI using NMR analysis (Fig. S8). Consistent with the ca. 2000-fold lower *k*_cat_/*K*_M_ value of PfpI *cf.* GloA1, when we compared the sensitivity of the Δ*pfpI* mutant and the isogenic wildtype progenitor to exogenously supplied MGO, there was no discernable difference (Fig. S9). Other explanations for this result are that the methylglyoxalase activity of PfpI is only manifested in certain growth conditions, or whereas MGO is a substrate of the enzyme, it is not the main physiological substrate.

Finally, we demonstrate that many of the phenotypes previously attributed to *pfpI*, based on analysis of PAO1-derived Tn mutants, were not reproducible in the Δ*pfpI* mutant. We used WGS analyses and found that the most likely origin of this disparity lies in the accrual of a large deletion in the Tn mutants and the presence of an SNP in *nalC*. Interestingly, we also found that the Tn mutant backgrounds supported a much higher level of expression of *pfpI* from a plasmid in *trans* than did the PAO1 progenitor. These findings highlight the importance of using WGS analyses to confirm mutant genetic backgrounds (57), and that complementation analyses, while informative, still have the potential to yield misleading results.

In summary, our data show that PfpI is a GSH-independent methylglyoxalase. This is important because all previous studies on the protein have assumed it to be a protease, based on the similarity of the protein with PfpI. However, in spite of looking for protease activity associated with the protein, we obtained no evidence for this. This highlights the dangers inherent in automated genome sequence annotation and stresses the ongoing importance of experimental investigation. Several gaps remain in the characterization of PfpI. For example, we have little understanding of what regulates its expression. Moreover, it is difficult to reconcile why expression of alternative GSH-dependent MGO detoxification pathways is downregulated in the Δ*pfpI* mutant. Also, we were unable to demonstrate whether glycated proteins or DNA (or RNA) are substrates of PfpI, so the kinetic parameters we report for MGO as a substrate may be underestimates of its true potential activity. Similarly, it is possible that MGO is just one substrate of the enzyme, and that its principle biological role is in detoxifying other dicarbonyls. Investigation of these issues is ongoing in the laboratory.

## Experimental procedures

### Bacterial strains and media

Unless otherwise stated, *P. aeruginosa* strains were routinely grown in Lennox lysogeny broth (LB) at 37 ˚C with vigorous aeration (shaking at 250 rpm). The strains used in this study are listed in Table S1.

### Cloning, overexpression, and purification of PfpI and YhbO

The ORF of *pfpI* (from *P. aeruginosa* PAO1) and *yhbO* (from *E. coli* DH5α) were PCR amplified from the genomic DNA of each organism using primer pairs PfpI OE Fwd/PfpI OE Rev and YhbO OE Fwd/YhbO OE Rev, respectively (Table S2). The PCR products were digested with NdeI and BamHI and then cloned into similarly digested pET-19m. This yielded constructs in which an N-terminal His_6_ tag was introduced onto each protein of interest. Cloning fidelity was confirmed following PCR amplification and sequencing across the MCS using primers pET-19m Fwd and pET-19m Rev. Protein expression was done using *E. coli* Rosetta (DE3) cells (Novagen). Cultures were grown in LB at 37 ˚C with good aeration until the midlog phase of growth. Protein expression was induced by the addition of isopropyl β-d-1-thiogalactopyranoside to a final concentration of 1 mM. The temperature was lowered to 16 ˚C, and growth was allowed to proceed for a further 16 h. The cells were then harvested by sedimentation at 10,000*g* for 20 min at four ˚C. The cell pellet was resuspended in 20 ml of buffer A (50 mM Tris–HCl, 300 mM NaCl, 5% [v/v] glycerol, 10 mM imidazole, pH 8.0), and the cells were ruptured by sonication (6 × 30 s, Soniprep 150, maximum power output). The lysate was clarified by centrifugation (20,000*g*, 40 min) followed by filtration through a 0.45 μm filter. The filtered supernatant was loaded onto a nickel–nitrilotriacetic acid (Qiagen) column pre-equilibrated with buffer A. The column was washed with buffer A, before elution with buffer B (50 mM Tris–HCl, 300 mM NaCl, 300 mM imidazole, 5% [v/v] glycerol). The eluted sample was dialyzed overnight against 4 l of buffer C (50 mM Tris–HCl, 100 mM NaCl, 5% [v/v] glycerol, pH 8.0) and concentrated by ultrafiltration in a 10,000 molecular weight cutoff centrifugal concentrator (Sartorius). Protein yields were determined by absorbance at 280 nm measurement of appropriately diluted samples. The calculated molecular mass and extinction coefficient (ε_280 nm_) for the His_6_-tagged PfpI were 21,246 Da and 24,980 M^−1^ cm^−1^, respectively. The corresponding values for His_6_-tagged YhbO were 20,858 Da and 4470 M^-1^ cm^-1^.

### Sedimentation velocity ultracentrifugation

Sedimentation velocity analytical ultracentrifugation was carried out using a Beckman Optima XL-I analytical ultracentrifuge (An-60 Ti rotor) fitted with absorbance and interference optical detection systems. The PfpI sample was prepared by dialyzing purified protein against 25 mM Tris–HCl (pH 8.0) and 100 mM NaCl to remove traces of glycerol. Epon double-sector centerpiece cells were loaded with a total of 400 μl of sample (protein) solution or buffer. Three different protein concentrations were tested (0.5, 1.0, and 2.0 mg ml^−1^). Sedimentation was done at 29,000*g* for 24 h at 20 ˚C. Buffer viscosity, protein partial specific volume, and frictional coefficient were calculated using SEDFIT (National Institutes of Health) (58). Data were analyzed using SEDNTERP (National Institutes of Health) (59).

### Protein crystallization

PfpI was crystallized using sitting drop vapor diffusion technique. Crystals were obtained after 10 days using 20 mg ml^-1^ PfpI mixed with an equal volume of 0.2 M calcium chloride, 0.1 M Tris–HCl (pH 8.5), and 20% (w/v) PEG 3350. The crystals were cryoprotected with 30% (v/v) glycerol and 70% (v/v) reservoir solution, mounted on nylon loops (Hampton Research), and flash frozen in liquid nitrogen in preparation for data collection.

### X-ray diffraction, structure determination, and refinement

Diffraction data were collected at the Diamond Light Source Synchrotron. The raw data were processed using Xia3 DIALS (60). Molecular replacement was done using Phaser (CCP4 suite). The apostructure of *P. horikoshii* PH1704 (PDB code: IG2I) was used as a model for phasing. Refmac (CCP4) and PHENIX.refine (Phenix) were used for automated structure refinement (61). Modeling and refinement was done using COOT (MRC Laboratory of Molecular Biology) (62). The PDB code for the refined structure is 8R3N (63). Crystallographic statistics are shown in Table S3.

### Generation of a Δ*pfpI* mutant

The Δ*pfpI* mutant in PAO1 was made using an overlap PCR-based approach, essentially as previously described (64). Briefly, regions, ca. 800 to 1000 bp upstream and downstream of the *pfpI* ORF were PCR amplified from PAO1 genomic DNA using primer pairs PfpI KO Up Fwd/PfpI KO Up Rev and PfpI KO Down Fwd/PfpI KO Down Rev, respectively (Table S2). The resulting (overlapping) amplicons were cloned into the suicide vector, pEX19Gm (64), using an NEBuilder HiFi DNA Assembly Cloning Kit (NEB). Cloning fidelity was confirmed following PCR amplification and sequencing across the MCS using primers pEX19Gm Fwd and pEX19Gm Rev. The confirmed plasmid was then introduced into *P. aeruginosa* by electroporation. Transformants were selected on LB-agar plates supplemented with 50 μg ml^−1^ gentamicin, and, following *sacB*-mediated selection on sucrose, colonies were screened for *pfpI* deletion by PCR. Deletion mutants were confirmed by PCR amplification across the junction region using primers PfpI KO check Fwd and PfpI KO check Rev and subsequent sequencing of the amplicon using primer PfpI KO GATC. Where indicated, the deletion mutant was complemented using *pfpI* cloned into pUCP20 (following PCR amplification of the *pfpI* ORF from PAO1 gDNA template using primers pUCP20-*pfpI* Fwd and pUCP20-*pfpI* Rev) (65). Cloning fidelity was confirmed following PCR amplification and sequencing across the MCS using primers pUCP20 Fwd and pUCP20 Rev.

### Antibody generation and Western blotting

Polyclonal rabbit antibodies (BioGenes, GmbH) were raised against purified recombinant PfpI and preadsorbed against an acetone extract of the *pfpI* deletion mutant before use. For Western blotting, proteins were resolved using a 12% (w/v) polyacrylamide SDS gel. The resolved proteins were transferred onto a polyvinylidene difluoride membrane, which was subsequently blocked using 5% (w/v) skimmed milk in PBS. The membranes were first probed with preadsorbed anti-PfpI antibodies and then with IRDye 800CW goat anti-rabbit IgG (LI-COR Biosciences). Labeled bands were detected using an Odyssey Infrared Imaging System (LI-COR Biosciences).

### NMR spectroscopy

Recombinant PfpI and YhbO were dialyzed against 50 mM PBS containing 5% (v/v) dimethyl sulfoxide-d_6_. The initial substrate concentration was set at 1 mM. Reactions were initiated by adding PfpI or YhbO (20 μg). Samples were incubated overnight at four ˚C and then loaded into 2.5 mm capillary inserts (New Era) for NMR data collection. Data were collected at 300 K on a Bruker AVANCE spectrometer operating at 500 MHz. Water suppression was achieved with low power presaturation and double-pulsed field gradient spin-echo excitation WATERGATE W5 (66, 67).

### Generation of site-directed C112 and H113 mutants

Three PfpI single mutants (C112A, H113A, and H113E) were generated using the Q5 Site-Directed Mutagenesis Kit (New England Biolabs) according to the manufacturer's protocol. The pET-19m expression vector containing the wildtype *pfpI* gene served as the template for mutagenesis. Briefly, primers were designed using the NEBaseChanger online tool (https://nebasechanger.neb.com/) to introduce the desired substitutions at the codons encoding C112 and H113 (Table S2). PCR amplification was then performed using the temperatures recommended by NEBaseChanger. Following ligation to reseal the plasmids, they were transformed into competent *E. coli* DH5α and grown in media containing 50 μg ml^-1^ carbenicillin (for selection of the plasmids). Sanger sequencing was performed using the universal pET-19M Fwd/pET-19M Rev primers (Table S2) to confirm the presence of the desired mutations. The plasmids were then introduced into Rosetta (DE3) (Novagen) for overexpression and purification of the PfpI mutant proteins. Mutant PfpI proteins were purified in the same way as the wildtype protein.

### Enzymatic activity

Purified PfpI activity was measured at 37 °C in PBS buffer. The substrate, MGO, was freshly prepared from 6.06 M stock to yield a 40 mM working stock solution in PBS. Reactions (50 μl volume) were initiated by adding 0.2 μM PfpI. At appropriate time intervals (0–35 min), the reaction was terminated by immersing the tubes into boiling water for 5 min. These samples were stored on ice for subsequent quantification of lactate using the protocol of Schmiedeknech *et al.* (38). The activity of the indicated PfpI mutants was measured using the same procedure but with a fixed concentration (200 μM) of MGO substrate. Kinetic parameters were calculated using GraphPad Prism 10 (GraphPad Software, Inc).

### Quantitative proteomic analysis

N = 4 independent cultures each of PAO1 and of the isogenic Δ*pfpI* mutant were grown in 50 ml Mops–glucose minimal media (40 mM Mops free acid, 4 mM tricine, 0.01 mM FeSO_4_, 9.52 mM NH_4_Cl, 0.5 μM CaCl_2_, 0.52 mM MgCl_2_, 50 mM NaCl, 0.29 mM K_2_SO_4_, 1.32 mM K_2_HPO_4_, 0.03 μM ammonium molybdate, 4 μM boric acid, 0.3 μM CoCl_2_, 0.1 μM CuSO_4_, 0.8 μM MnCl_2_, 0.1 μM ZnSO_4_, 20 mM d-(+)-glucose) with good aeration until absorbance of 0.8 to 0.9 at 600 nm. Cell pellets were resuspended in 0.5 ml 100 mM Tris–HCl, 50 mM NaCl, 10% (v/v) glycerol, 1 mM *tris*(2-carboxyethyl)phosphine (TCEP), pH 7.5. Samples were sonicated 3 × 5 s on ice, and the lysates were clarified by sedimentation at 21,000*g* for 45 min at 4 ˚C. Protein concentration was determined using the DC protein assay (Bio-Rad) and adjusted to 2 mg ml^−1^. Proteolytic digestion, reduction, alkylation, tandem mass tag labeling, peptide fractionation, and peptide quantification (LC–MS/MS) were carried out essentially as previously described (68) at the Cambridge Centre for Proteomics. Proteome Discoverer v2.1 (Thermo Fisher Scientific) and Mascot v2.6 (Matrix Science) were used to align the quantified peptides against the UniProt *P. aeruginosa* database and the common repository of adventitious proteins (cRAP), version 1.0. Comparative analyses were carried out using “R” software. Additional data processing was done using MSnbase (69), including missing value removal, log_2_ transformation, and sample normalization. Differential abundance of protein was determined using the Limma package (70). The statistical significance of differences in abundance was determined using Student's *t* test with variances moderated by Limma's empirical Bayes method. All *p* values were adjusted for multiple testing using the Benjamini–Hochberg method (71). In this study, modulations with a log_2_ fold change of >1 or <-1 and false discovery rate–adjusted *p* value of ≤0.05 were considered statistically significant.

### Whole-genome sequencing

WGS of PAO1_MW(LG)_ (the specific wildtype progenitor used in the current study; the subscript is used to indicate exactly which PAO1 lineage is being used) (57), Δ*pfpI*, PW1654, and PW1655 was carried out using a combination of Illumina and Oxford Nanopore sequencing platforms by MicrobesNG (Enhanced Genome Service). Reads were trimmed with Trimmomatic and mapped to the *P. aeruginosa* PAO1 reference genome (72). Samtools, Bedtools, and the Burrows–Wheeler Aligner (MEM) software packages were used to assess data quality (73, 74, 75). *De novo* assembly of the reads was done using SPAdes. Genome assemblies (FASTA format) were interrogated using Artemis. Variant calling and contig annotation were done using VarScan and Prokka, respectively (76).

### Growth kinetics

Bacterial growth was monitored in microtiter plates using a BMG Labtech FLUOstar Omega microplate reader. Briefly, *P. aeruginosa* overnight cultures were subcultured and allowed to reach an absorbance of 1.0 at 595 nm. These starter cultures were then used to inoculate 100 μl of the indicated medium to a starting absorbance of 0.05 at 595 nm in flat-bottomed microtiter plates (Greiner Bio-One). The plates were sealed with a gas permeable membrane (Breathe-Easy) and incubated in the plate reader at 37 ˚C with rotary agitation at 250 rpm. Four readings per well were taken every 15 min at a wavelength of 595 nm. Measurements were made on triplicate cultures of each strain. Statistical significance was determined using an unpaired *t* test.

### Swarming motility

Swarm agar contained (per liter): 1.07 g NH_4_Cl, 1.7 g Na_2_HPO_4_, 3.0 g KH_2_PO_4_, 0.5 g NaCl, 1.98 g dextrose, 5 g casein hydrolysate, and 5 g bacteriological grade granulated agar (Formedium). After autoclaving, MgSO_4_ and CaCl_2_ were added to a final concentration of 1 mM and 0.1 mM, respectively. Overnight cultures were normalized to an absorbance of 1.0 at 595 nm, and 2.5 μl were spotted onto the agar surface. Plates were incubated at 37 ˚C for 8 to 18 h before recording.

### Ciprofloxacin resistance

MICEvaluators (Oxoid; catalog no.: MA0104) were used to determine the MIC of ciprofloxacin, as per the manufacturer's instructions.

## Data availability

The whole-genome sequence data have been deposited in the National Center for Biotechnology Information server (Bioproject accession no.: PRJNA1028845). The proteomics data have been deposited in the ProteomeXchange Consortium *via* the PRIDE partner repository (accession no.: PXD046055). The PDB code for the refined structure is 8R3N.

## Supporting information

This article contains supporting information, including a list of bacterial strains (Table S1) and primers (Table S2) used, crystallographic statistics (Table S3), list of ORFs affected by deletions in PW1654 and PW1655 (Table S4), area under the ROC curve data on recombinant PfpI (Fig. S1), and NMR confirmation ([^1^H] NMR and [^13^C] NMR spectra and COSY, heteronuclear single quantum coherence, and heteronuclear multiple bond correlation analyses) of lactate as reaction product (Figs. S2, S3), ciprofloxacin sensitivity of *pfpI* mutants (Fig. S4), twin-*Strep* tag pull-down analyses (Fig. S5), swarming phenotype of *pfpI* mutants (Fig. S6), H_2_O_2_ sensitivity of *pfpI* mutants (Fig. S7), NMR confirmation ([^1^H] NMR) of glyoxal conversion to glycolate by PfpI (Fig. S8), and MGO sensitivity of Δ*pfpI* mutant *versus* wildtype (Fig. S9) (20, 64, 65, 68).

## Conflict of interest

The authors declare that they have no conflicts of interest with the contents of this article.

## Supporting information

Proteomics SI file Peptide groups

Proteomics SI file Proteins
