# Supplementary material for: Pseudomonas aeruginosa PfpI is a methylglyoxalase
Source: J Biol Chem. 2025 Mar 3;301(4):108374. doi: 10.1016/j.jbc.2025.108374 (PMC11987610; doi:10.1016/j.jbc.2025.108374)
Supplement: Supporting Information [file mmc4.pdf]

Supporting Information

Table S1. Bacterial strains and plasmids.

Strain	Genotype or relevant characteristics	Source
P. aeruginosa		
PAO1	Chloramphenicol (Cam) resistant *Pseudomonas aeruginosa* isolate originally derived from a human wound	Jacobs *et al*., 2003
PAO1 Δ*pfpI*	PAO1 with in-frame deletion of *pfpI*	This study
PW1654	PAO1 harbouring a transposon (ISlacZ/hah) insertion in *pfpI*, Tet^R	Jacobs *et al*., 2003
PW1655	PAO1 harbouring a transposon (IS*phoA*/hah) insertion in *pfpI*, Tet^R	Jacobs *et al*., 2003
PAO1-Strep-PfpI	PAO1 with twin-*Strep*-tag fused to N-terminus of chromosomal copy of *pfpI*	This study
E. coli		
Rosetta™ 2 BL21(DE3)	F^- *ompT hsdS*$_B$(r_B^- m_B^-) *gal dcm* (DE3) pRARE2 (Cam^R)	Novagen
DH5α	*rec*A1-, *end*A-deficient chemicompetent cells	Thermo Fisher Scientific
DE3-YhbO	Rosetta™ 2 BL21(DE3) carrying pET-19m-*yhbO*, $Carb^R$, Cam^R	This study
DE3-PfpI	Rosetta™ 2 BL21(DE3) carrying pET-19m-*pfpI*, $Carb^R$, Cam^R	This study
DE3-PfpI-C112A	Rosetta™ 2 BL21(DE3) carrying pET-19m-*pfpI*-C112A, $Carb^R$, Cam^R	This study
DE3-PfpI-H113A	Rosetta™ 2 BL21(DE3) carrying pET-19m-*pfpI*-H113A, $Carb^R$, Cam^R	This study
DE3-PfpI-H113E	Rosetta™ 2 BL21(DE3) carrying pET-19m-*pfpI*-H113E, $Carb^R$, Cam^R	This study
**Plasmids**		
pUCP20	*Escherichia* to *Pseudomonas* shuttle vector, $Carb^R$	West *et al*., 1994
pUCP20-*pfpI*	pUCP20 shuttle vector containing the ORF and upstream, native promoter of *pfpI*, $Carb^R$	This study
pET-19m	Vector used for inducible overexpression of N-terminal His_6-tagged proteins, $Carb^R$	Dolan *et al*., 2022
pET-19m-*pfpI*	Vector for inducible overexpression of N-terminal His_6-tagged *pfpI*, $Carb^R$	
pET-19m-*yhbO*	Vector for inducible overexpression of N-terminal His_6-tagged *yhbO*, $Carb^R$	This study
pET-19m-*pfpI*-C112A	Identical to 'pET19m-*pfpI*', except for C112A mutation	This study

pET-19m-*pfpI*-H113A	Identical to ‘pET19m-*pfpI*’, except for H113A mutation	This study
pET-19m-*pfpI*-H113E	Identical to ‘pET19m-*pfpI*’, except for H113E mutation	This study
pEX19Gm	*oriT*, *sacB*, *lacZα*, MCS from pUC19, Gm^R	Huang *et al*., 2017
pEX19Gm-*pfpI*	*oriT*, *sacB*, *lacZα*, fusion protein consisting of *pfpI* flanking regions, Gm^R	This study

Table S2. List of primers. For the primers used for site-directed mutagenesis, the altered bases are shown in lower case.

Primer	Sequence (5' – 3')	Restriction site
PfpI OE Fwd	ATATACTACATATGATGACCCAATCCCTGCACG	NdeI
PfpI OE Rev	ATATGGATCCTCAGCCGGCGAGGATTTCG	BamHI
YhbO OE Fwd	ATACAGTACATATGATGAGTAAGAAAATTGCCGTTT TAATC	NdeI
YhbO OE Rev	ATATGGATCCTCAGGCACCGAGCAGGCG	BamHI
pET-19m Fwd	GGATATAGTTCCTCCTTTCAGC	N/A
pET-19m Rev	GTAGAGGATCGAGATCTCGATC	N/A
pUCP20-*pfpI* Fwd	TGACAAGCTTCATCTGCAGGTCGACCATG	HindIII
pUCP20-*pfpI* Rev	TCAGGGATCCTCAGCCGGCGAGGATTTCG	BamHI
pUCP20 Fwd	CTCTTCGCTATTACGCCAGC	N/A
pUCP20 Rev	CTGGAAAGCGGGCAGTGAG	N/A
PfpI KO Up Fwd	ACCCGGGGATCCTCTTCTTCAGTTGGGTATCGCG	N/A
PfpI KO Up Rev	GGGATGTCGTGCAGGGATTGGGTCATG	N/A
PfpI KO Down Fwd	CCCTGCACGACATCCCGGCCTTCAAC	N/A
PfpI KO Down Rev	CTGCAGGTCGACTCTTTCTTGCGCTTGATGAAGG	N/A
pEX19Gm Fwd	TTGTAAAACGACGGCCAGTG	N/A
pEX19Gm Rev	TTGTGTGGAATTGTGAGCGG	N/A
PfpI KO check Fwd	CAGCTCGATGTCGACGTGC	N/A
PfpI KO check Rev	CAGGTCAGGCTCTTGATGTAG	N/A
PfpI KO GATC	CAGGCTCAGGCAGCTCATG	N/A
PfpI-C112A Fwd	GGCGGTGATCgccCACGGCGCCTG	N/A
PfpI-C112A Rev	ACCGGCTTGCTCGCCTGT	N/A
PfpI-H113A Fwd	GGTGATCTGCgccGGCGCCTGGCTGC	N/A
PfpI-H113A Rev	GCCACCGGCTTGCTCGCC	N/A
PfpI-H113E Fwd	GGTGATCTGCgaaGGCGCCTGGCTG	N/A
PfpI-H113E Rev	GCCACCGGCTTGCTCGCC	N/A

Table S3. Crystallographic statistics.

Structure	PfpI (PDB: 8R3N)
**Data Collection**	
Wavelength (Å)	0.9795
Resolution range (Å)	39.35 - 1.45 (1.502 - 1.45)
Space group	P 2_1 2_1 2_1
Unit cell	
a, b, c (Å)	41.99 78.71 103.09
a, b, g (°)	90 90 90
Total reflections	365039 (6258)
Unique reflections	59398 (2343)
Multiplicity	6.1 (2.7)
Completeness (%)	96.47 (81.07)
Mean I/sigma(I)	11.9 (0.9)
Wilson B-factor	18.82
R-meas	0.081 (1.158)
CC1/2	1 (0.6)
**Refinement**	
Resolution range (high resolution) (Å)	39.35 - 1.45 (1.502 - 1.45)
Reflections used in refinement	59232 (4899)
Reflections used for R-free	2966 (265)
R-work	0.2546 (0.3991)
R-free	0.2729 (0.4429)
Number of non-hydrogen atoms	3079
Macromolecules	2723
Ligand	0
Solvent	356
Protein residues	356
RMS (bonds) Å	0.009
RMS (angles) ()	0.88
Ramachandran favoured (%)	98.01
Ramachandran allowed (%)	1.99
Ramachandran outliers (%)	0.00
Rotamer outliers (%)	0.35
Clashscore	2.95
Average B-factor	20.95
Macromolecules	20.04
Ligands	-
Solvent	27.90

Table S4. Deletions identified by whole genome sequencing analyses of PAO1 *pfpI* transposon mutants. Note, if a column entry under the heading "PW1654" or "PW1655" is blank, this means that the locus is present. For example, on line 1 of the main body of the table, the locus PA2395 (*pvdO*) is present in PW1654 but has been deleted in PW1655. Emboldened loci are absent in both PW1654 and PW1655.

PW1654	PW1655	Gene name	Description
	PA2395	*pvdO*	
	PA2396	*pvdF*	Pyoverdine synthetase F
	PA2397	*pvdE*	Pyoverdine biosynthetic protein E
	PA2398	*fpvA*	Ferripyoverdine receptor
	PA2399	*pvdD*	Pyoverdine synthetase D
	PA2400	*pvdJ*	
**PA2402**	**PA2402**	***pvdI***	Pyoverdine peptide synthetase
**PA2403**	**PA2403**	***fpvG***	
**PA2404**	**PA2404**	***fpvH***	
**PA2405**	**PA2405**	***fpvJ***	
**PA2406**	**PA2406**	***fpvK***	
**PA2407**	**PA2407**	***fpvC***	
**PA2408**	**PA2408**	***fpvD***	
**PA2409**	**PA2409**	***fpvE***	
**PA2410**	**PA2410**	***fpvF***	
**PA2411**	**PA2411**		Probable thioesterase
**PA2412**	**PA2412**		Conserved hypothetical protein
**PA2413**	**PA2413**	***pvdH***	L-2,4-diaminobutyrate:2-ketoglutarate 4-aminotransferase
**PA2414**	**PA2414**		L-sorbosone dehydrogenase
**PA2415**	**PA2415**		Hypothetical protein
**PA2416**	**PA2416**	***treA***	Periplasmic trehalase precursor
**PA2417**	**PA2417**		Probable transcriptional regulator
**PA2418**	**PA2418**		Hypothetical protein
**PA2419**	**PA2419**		Probable hydrolase
**PA2420**	**PA2420**		Probable porin
**PA2421**	**PA2421**		Hypothetical protein
**PA2422**	**PA2422**		Hypothetical protein
**PA2423**	**PA2423**		Hypothetical protein
**PA2424**	**PA2424**	***pvdL***	
**PA2425**	**PA2425**	***pvdG***	
**PA2426**	**PA2426**	***pvdS***	Sigma factor
**PA2427**	**PA2427**		Hypothetical protein
**PA2428**	**PA2428**		Hypothetical protein
**PA2429**	**PA2429**		Hypothetical protein
**PA2430**	**PA2430**		Conserved hypothetical protein

**PA2431**	**PA2431**		Hypothetical protein
**PA2432**	**PA2432**	***bexR***	Bistable expression regulator
**PA2433**	**PA2433**		Hypothetical protein
**PA2434**	**PA2434**		Hypothetical protein
**PA2435**	**PA2435**		Probable cation-transporting P-type ATPase
**PA2436**	**PA2436**		Hypothetical protein
**PA2437**	**PA2437**		Hypothetical protein
**PA2438**	**PA2438**		Hypothetical protein
**PA2439**	**PA2439**		Hypothetical protein
**PA2440**	**PA2440**		Hypothetical protein
**PA2441**	**PA2441**		Hypothetical protein
**PA2442**	**PA2442**	***gcvT2***	Glycine cleavage system protein T2
**PA2443**	**PA2443**	***sdaA***	L-serine dehydratase
**PA2444**	**PA2444**	***glyA2***	Serine hydroxymethyltransferase
**PA2445**	**PA2445**	***gcvP2***	Glycine cleavage system protein P2
**PA2446**	**PA2446**	***gcvH2***	Glycine cleavage system protein H2
**PA2447**	**PA2447**		Probable transcriptional regulator
**PA2448**	**PA2448**		Putative hydrolase
**PA2449**	**PA2449**	***gcsR***	Glycine cleavage system regulator
**PA2450**	**PA2450**		Hypothetical protein
**PA2451**	**PA2451**		Hypothetical protein
**PA2452**	**PA2452**		Hypothetical protein
**PA2453**	**PA2453**		Hypothetical protein
**PA2454**	**PA2454**		Hypothetical protein
**PA2455**	**PA2455**		Hypothetical protein
**PA2456**	**PA2456**		Hypothetical protein
**PA2457**	**PA2457**		Hypothetical protein
**PA2458**	**PA2458**		Hypothetical protein
**PA2459**	**PA2459**		Hypothetical protein
**PA2460**	**PA2460**		Hypothetical protein
**PA2461**	**PA2461**		Hypothetical protein
**PA2462**	**PA2462**		Hypothetical protein
**PA2463**	**PA2463**		Hypothetical protein
**PA2464**	**PA2464**		Hypothetical protein
**PA2465**	**PA2465**		Hypothetical protein
**PA2466**	**PA2466**	***foxA***	Ferrioxamine receptor
**PA2467**	**PA2467**	***foxR***	Anti-sigma factor
**PA2468**	**PA2468**	***foxI***	ECF sigma factor
**PA2469**	**PA2469**		Probable transcriptional regulator
**PA2470**	**PA2470**	***gtdA***	Gentisate 1,2-dioxygenase
**PA2471**	**PA2471**		Conserved hypothetical protein
**PA2472**	**PA2472**		Probable major facilitator superfamily (MFS) transporter
**PA2473**	**PA2473**		Maleylpyruvate isomerase
**PA2474**	**PA2474**		Hypothetical protein
**PA2475**	**PA2475**		Probable cytochrome P450

**PA2476**	**PA2476**	***dsbG***	Thiol:disulfide interchange protein
**PA2477**	**PA2477**	***dsbE***	Thiol:disulfide interchange protein
**PA2478**	**PA2478**	***dsbD***	Thiol:disulfide interchange protein
**PA2479**	**PA2479**	***dsbR***	Dsb-associated regulator
**PA2480**	**PA2480**	***dsbS***	Dsb-associated sensor
**PA2481**	**PA2481**		Hypothetical protein
**PA2482**	**PA2482**		Probable cytochrome c
**PA2483**	**PA2483**		Conserved hypothetical protein
**PA2484**	**PA2484**		Conserved hypothetical protein
**PA2485**	**PA2485**		Hypothetical protein
**PA2486**	**PA2486**	***ptrC***	Pseudomonas type III repressor gene
**PA2487**	**PA2487**		Hypothetical protein
**PA2488**	**PA2488**		Probable transcriptional regulator
**PA2489**	**PA2489**		Probable transcriptional regulator
**PA2490**	**PA2490**		Conserved hypothetical protein
**PA2491**	**PA2491**	***mexS***	Probable oxidoreductase
**PA2492**	**PA2492**	***mexT***	Transcriptional regulator
**PA2493**	**PA2493**	***mexE***	RND multidrug efflux membrane fusion protein precursor
**PA2494**	**PA2494**	***mexF***	RND multidrug efflux transporter
**PA2495**	**PA2495**	***oprN***	Multidrug efflux outer membrane protein precursor
**PA2496**	**PA2496**		Hypothetical protein
**PA2497**	**PA2497**		Probable transcriptional regulator
**PA2498**	**PA2498**		Conserved hypothetical protein
**PA2499**	**PA2499**		Probable deaminase
**PA2500**	**PA2500**		Probable major facilitator superfamily (MFS) transporter
**PA2501**	**PA2501**		Hypothetical protein
**PA2502**	**PA2502**		Hypothetical protein
**PA2503**	**PA2503**		Hypothetical protein
**PA2504**	**PA2504**		TUDOR-like domain containing protein
**PA2505**	**PA2505**	***opdT***	Tyrosine porin
**PA2506**	**PA2506**		Hypothetical protein
**PA2507**	**PA2507**	***catA***	Catechol 1,2-dioxygenase
**PA2508**	**PA2508**	***catC***	Muconolactone delta-isomerase
**PA2509**	**PA2509**	***catB***	Muconate cycloisomerase I
**PA2510**	**PA2510**	***catR***	Transcriptional regulator
**PA2511**	**PA2511**	***antR***	Transcriptional regulator
**PA2512**	**PA2512**	***antA***	Anthranilate dioxygenase, large subunit
**PA2513**	**PA2513**	***antB***	Anthranilate dioxygenase, small subunit
**PA2514**	**PA2514**	***antC***	Anthranilate dioxygenase reductase
**PA2515**	**PA2515**	***xylL***	Cis-1,2-dihydroxycyclohexa-3,4-diene carboxylate dehydrogenase
**PA2516**	**PA2516**	***xylZ***	Toluate 1,2-dioxygenase electron transfer component
**PA2517**	**PA2517**	***xylY***	Toluate 1,2-dioxygenase beta subunit
**PA2518**	**PA2518**	***xylX***	Toluate 1,2-dioxygenase alpha subunit
**PA2519**	**PA2519**	***xylS***	Transcriptional regulator

**PA2520**	**PA2520**	***czcA***	RND divalent metal cation efflux transporter
PA2523		*czcR*	Copper-responsive two-component system regulator
PA2524		*czcS*	Copper-responsive two-component system sensor
PA2525		*opmB*	RND-type multidrug efflux pump system component
PA2526		*muxC*	RND-type multidrug efflux pump system component
PA2527		*muxB*	RND-type multidrug efflux pump system component
PA2528		*muxA*	RND-type multidrug efflux pump system component
PA2529			Hypothetical protein
PA2530			Hypothetical protein
PA2531			Probable aminotransferase
PA2532		*tpx*	Thiol peroxidase
PA2533			Probable sodium:alanine symporter
PA2534			Probable transcriptional regulator
PA2535			Probable oxidoreductase
PA2536			Probable phosphatidate cytidylyltransferase
PA2537			Probable acyltransferase
PA2538			Hypothetical protein
PA2539			Conserved hypothetical protein

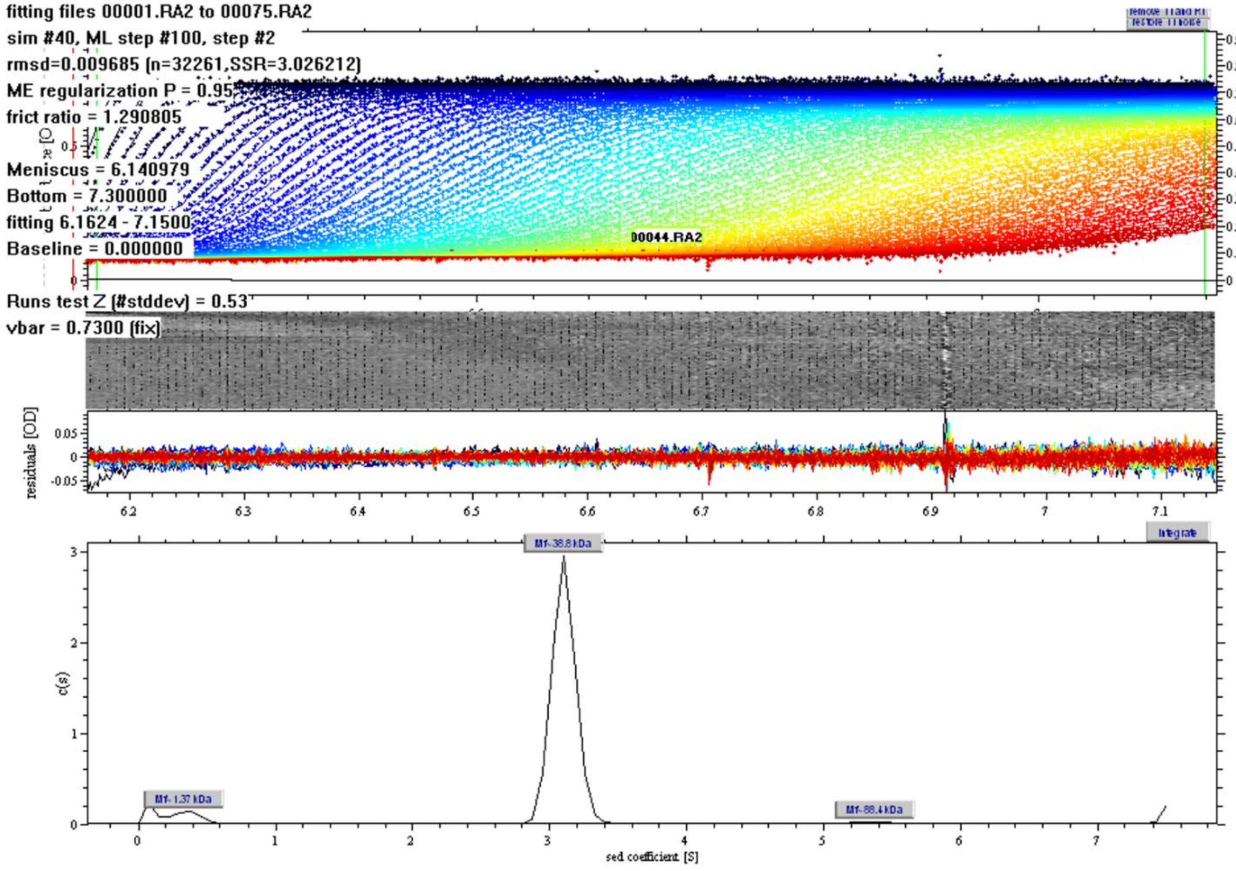

Figure S1. **Sedimentation velocity-AUC of recombinant *P. aeruginosa* PfpI.** The figure shows 100 absorbance scans of a PfpI protein sample (0.5 mg mL$^{-1)}$ recorded at a wavelength of 280 nm. The RMSD of the scans was 0.0097. The most prominent peak observed corresponds to an apparent molecular mass of 38.8 kDa, indicating that PfpI is most likely a dimer in solution. The frictional ratio of 1.29 suggests that the dimer is a slightly extended globule.

(A) [^{1}H] NMR (D_2O, 500 MHz)

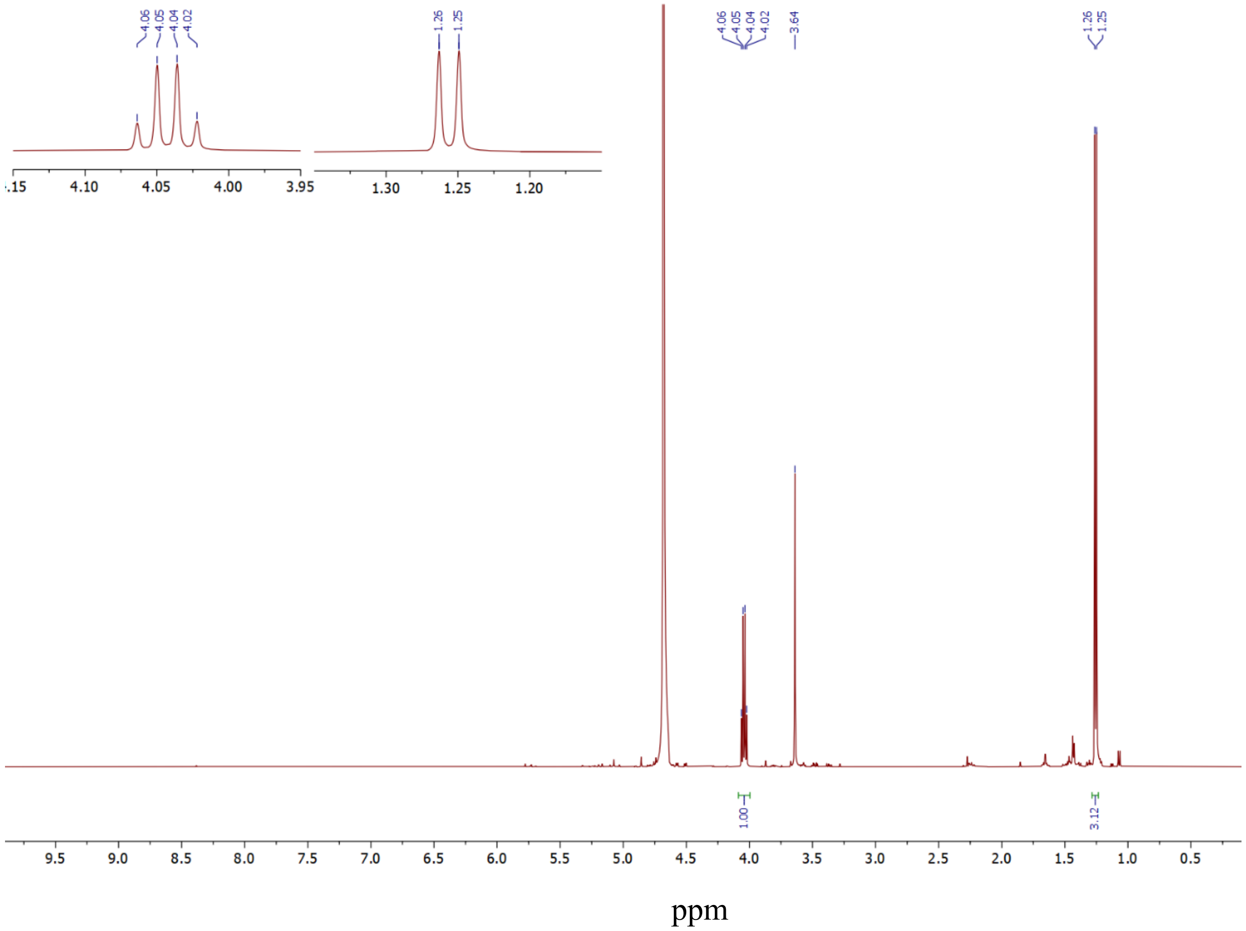

(B) [^{13}C] NMR (D_2O, 126 MHz)

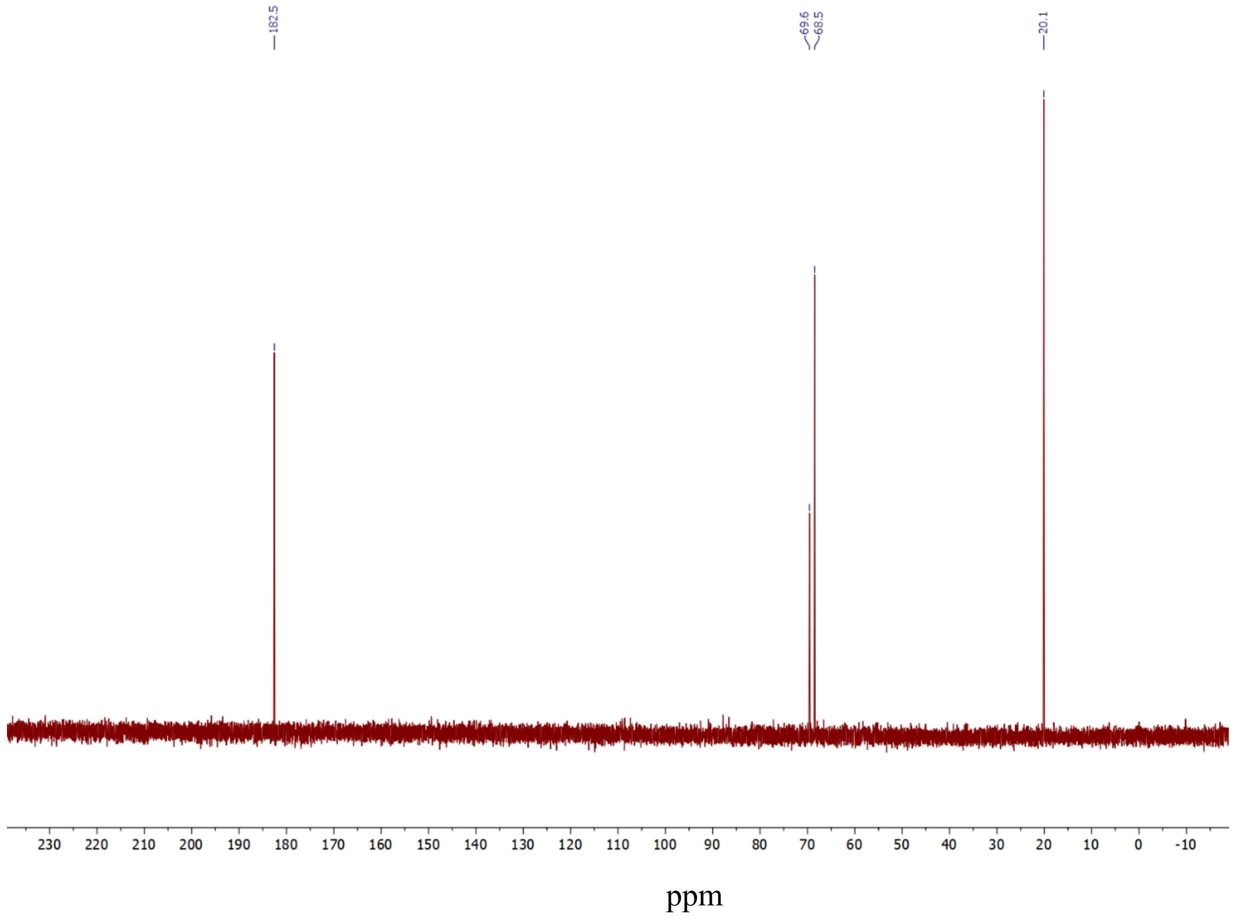

(C) ^{1}H-^{1}H Correlation Spectroscopy (COSY)

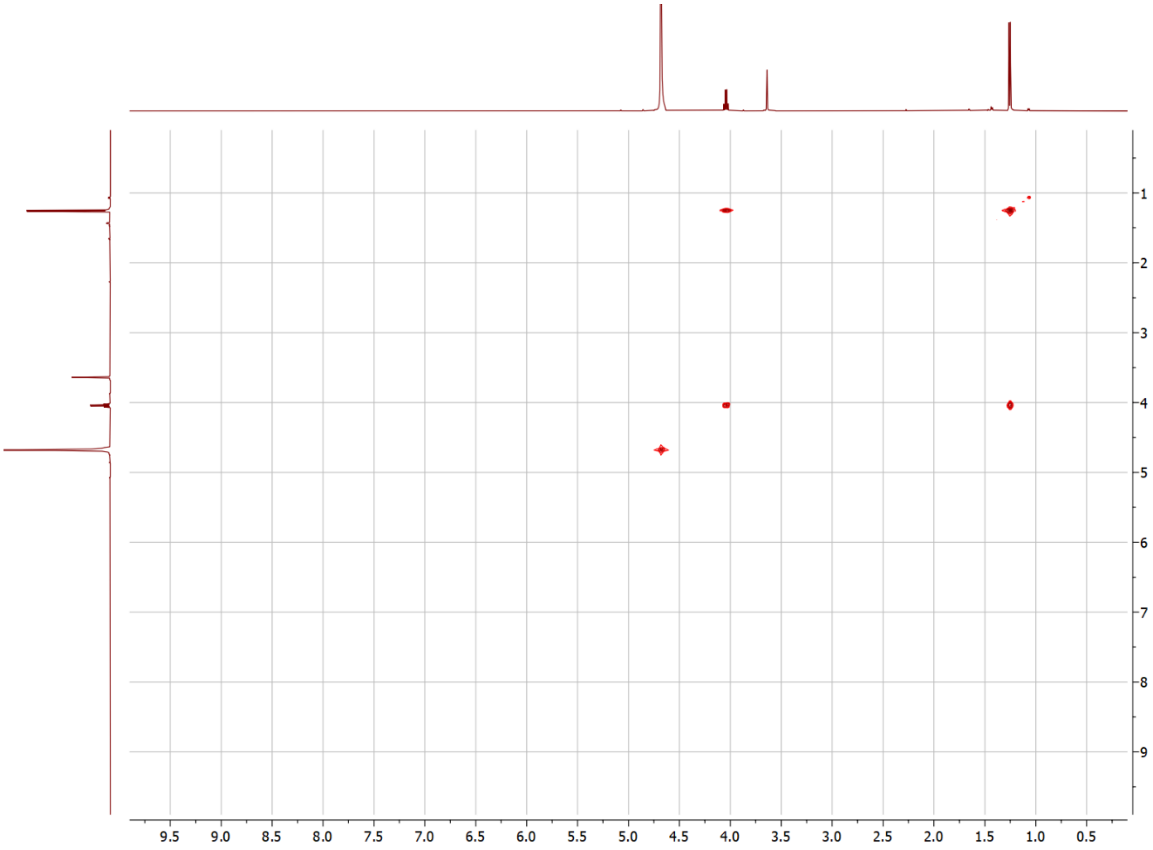

(D) Heteronuclear Single Quantum Coherence (HSQC)

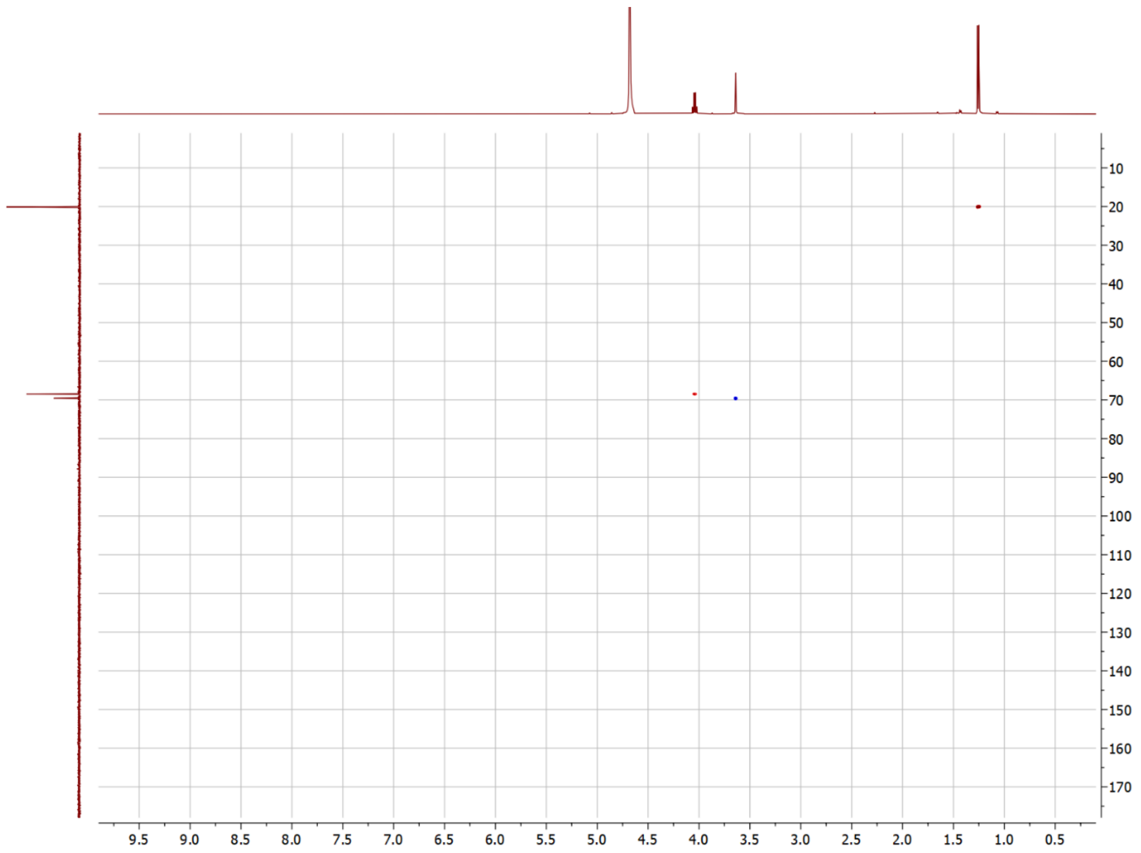

(E) Heteronuclear Multiple Bond Correlation (HMBC)

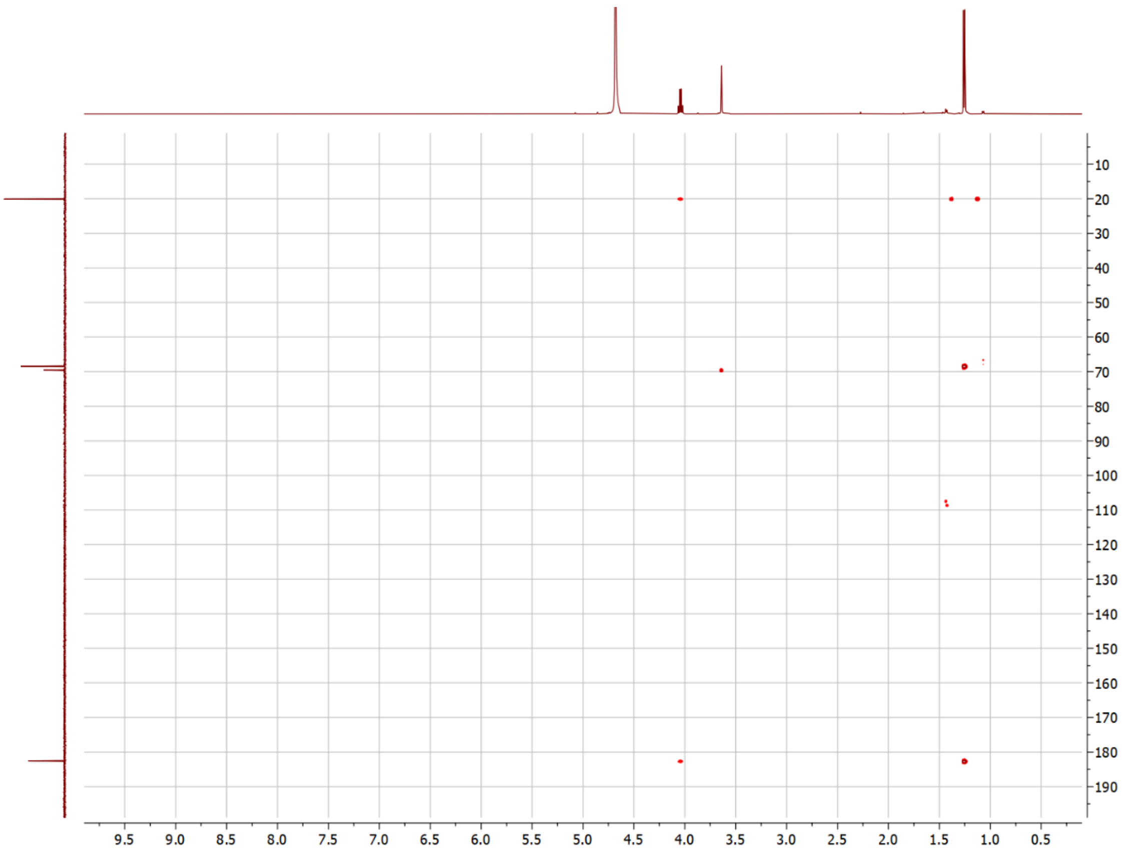

Figure S2. Analysis of the reaction product by NMR. Purified PfpI (20 μg) was mixed with MGO (1 mM) and incubated overnight at 4˚C. The sample was then lyophilized and resuspended in D_2O for NMR analysis. **(A)** [^{1}H] NMR (D_2O, 500 MHz); **(B)** [^{13}C] NMR (D_2O, 126 MHz); **(C)** ^{1}H-^{1}H Correlation Spectroscopy (COSY); **(D)** Heteronuclear Single Quantum Coherence (HSQC); and **(E)** Heteronuclear Multiple Bond Correlation (HMBC). The data are all consistent with the product of the PfpI/YhbO-catalyzed reaction being lactic acid. Peaks corresponding to lactic acid are as follows: [^{1}H] NMR (D_2O, 500 MHz) δ 4.04 (1H, q, J = 6.9 Hz, C*H*(OH)), 1.26 (3H, d, J = 6.9 Hz, C*H*3); [^{13}C] NMR (D_2O, 126 MHz) δ 182.5 (*C*O_2H), 68.5 (*C*H(OH)), 20.1 (*C*H_3). The additional peaks with chemical shifts of [^{1}H] NMR δ 3.64 (s) and [^{13}C] NMR δ 69.6 correspond to

ethylene glycol. The COSY spectrum indicates that nuclei with chemical shifts of δ_H 4.04 ppm and 1.26 ppm are on adjacent carbon atoms. The HSQC spectrum shows that the H atoms with chemical shifts of δ_H 4.04 ppm and 1.26 ppm are separated by a single bond, and that the carbon atoms display chemical shifts of δ_C 68.5 ppm and 20.1 ppm, respectively. The HSQC spectrum indicates the carbon atom with the chemical shift of δ_C 182.5 ppm is in close proximity to H atoms with chemical shifts of δ_H 4.04 ppm and 1.26 ppm.

(A) [^{1}H] NMR (D_2O, 500 MHz)

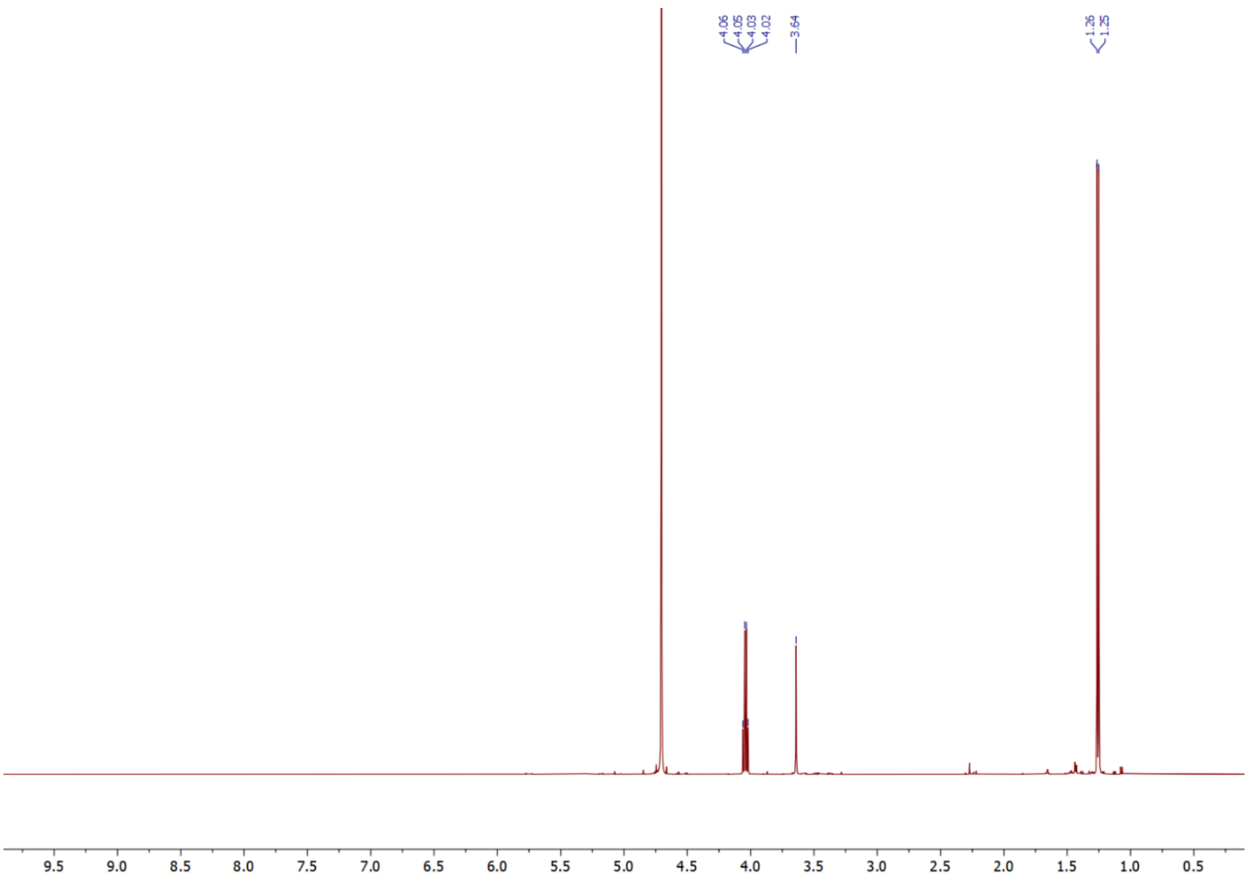

(B) [^{13}C] NMR (D_2O, 126 MHz)

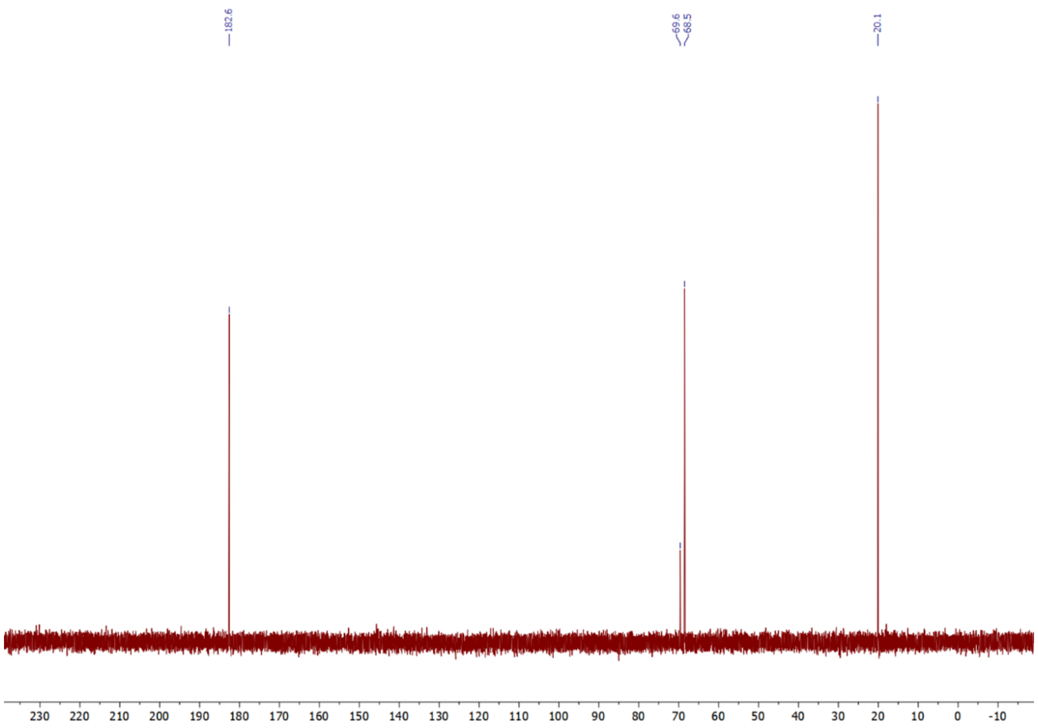

Figure S3. **Spiking the reaction end products with lactate did not lead to any new peaks appearing in the NMR spectra**. **(A)** [^{1}H] NMR (D_2O, 500 MHz) and **(B)** [^{13}C] NMR (D_2O, 126 MHz). Compared with the [^{1}H] NMR and [^{13}C] NMR spectrum displayed in **Figure S2(A)** and **S2(B)**, adding sodium lactate does not alter the appearance of the spectrum. These observations further confirm that the product of PfpI/YhbO action on MGO is indeed lactic acid. In this experiment, 13 mg of the lyophilized reaction product (which corresponds to approximately 1 mg metabolite, with the remainder being salts derived from the PBS) were dissolved in D_2O containing 1 mg racemic sodium lactate before analysis using NMR. Note that the L- and D-lactate moieties are indistinguishable by NMR.

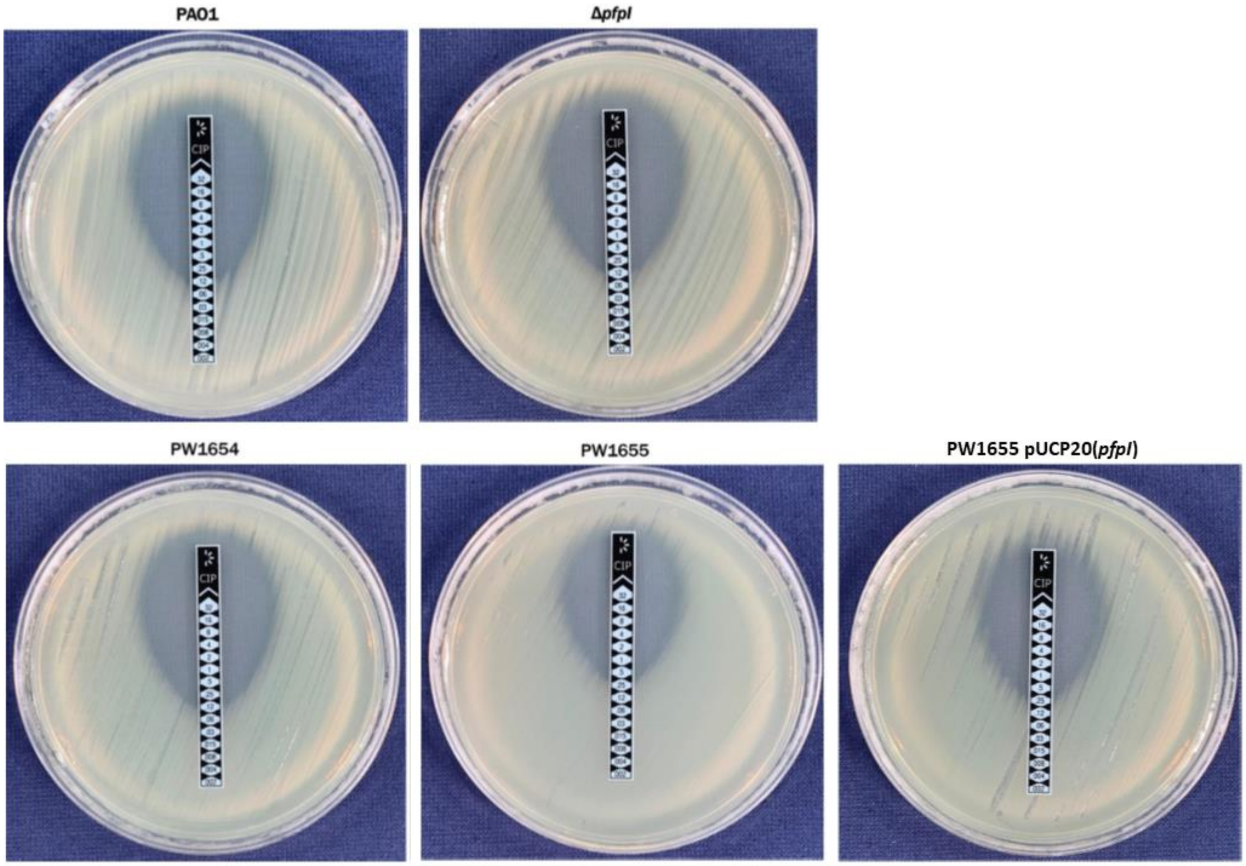

Figure S4. **$MIC_{ciprofloxacin}$ determination for PAO1, the Δ*pfpI* mutant (this study) and the UWGC Tn mutants, PW1654 and PW1655**. MIC vales were determined using ciprofloxacin evaluator (Oxoid) test strips, as indicated. The MIC (in μg/mL) corresponds to the point at which the clear zone intersects with the evaluator strip. Each evaluation was carried out on three separate replicates. Representative data are shown. Briefly, overnight cultures of the indicated strains were diluted to OD_{600} = 1.0 and then swabbed onto Müller-Hinton Agar (MHA) plates. The plates were incubated at room temperature for 5 min before applying the MIC evaluator strip. The plates were incubated overnight at 37˚C before reading. Note the increased resistance of PW1655 to ciprofloxacin (*cf*. PAO1, the Δ*pfpI* mutant, and PW1654), and that this elevated resistance is not complemented by provision of *pfpI in trans* on pUCP20.

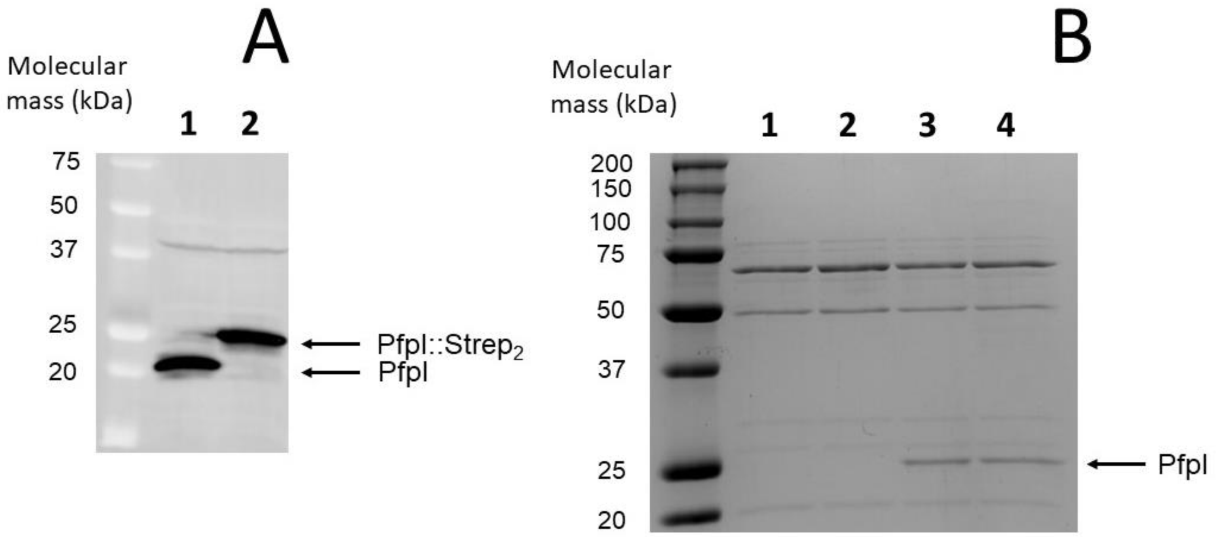

Figure S5. **PfpI does not interact with other proteins.** (**A**) **Expression of chromosomally-encoded twin-*Strep*-tagged PfpI is the same as that of untagged PfpI**. The chromosomal copy of *pfpI* in PAO1 was engineered to encode a twin-*Strep*-tag inserted at the N-terminus. Polyclonal anti-PfpI antibodies were used to detect PfpI and twin-*Strep*-tagged PfpI in cell extracts of PAO1 alone (lane 1) or in extracts of PAO1 encoding the twin-*Strep*-tagged PfpI (lane 2). Note the similar expression levels of tagged and untagged PfpI. The twin-*Strep*-tagged PfpI has a slightly higher molecular mass due to the presence of the twin-Strep-tag. (**B**) **Twin-*Strep*-tagged PfpI does not interact with other proteins**. The panel shows a Coomassie-stained gel of the proteins eluted from streptavidin beads following resolution of the eluted proteins by SDS-PAGE. Lanes 1 and 2; pulldowns from PAO1 expressing untagged PfpI, lanes 3 and 4; pulldowns from PAO1 expressing twin-*Strep*-tagged PfpI. Lanes 1 and 3; Cells lysed in the absence of 0.5% Triton X-100, lanes 2 and 4; cells were lysed in the presence of 0.5% Triton X-100. Note that apart from twin-*Strep*-tagged PfpI itself in lanes 3 and 4, the only proteins that are pulled down with the Streptavidin beads – irrespective of the stringency of the pulldown conditions (i.e., ± Triton-X100) - are also present in the control lanes (1 and 2) which do not contain twin-*Strep*-tagged protein. We conclude that PfpI does not interact strongly with other proteins.

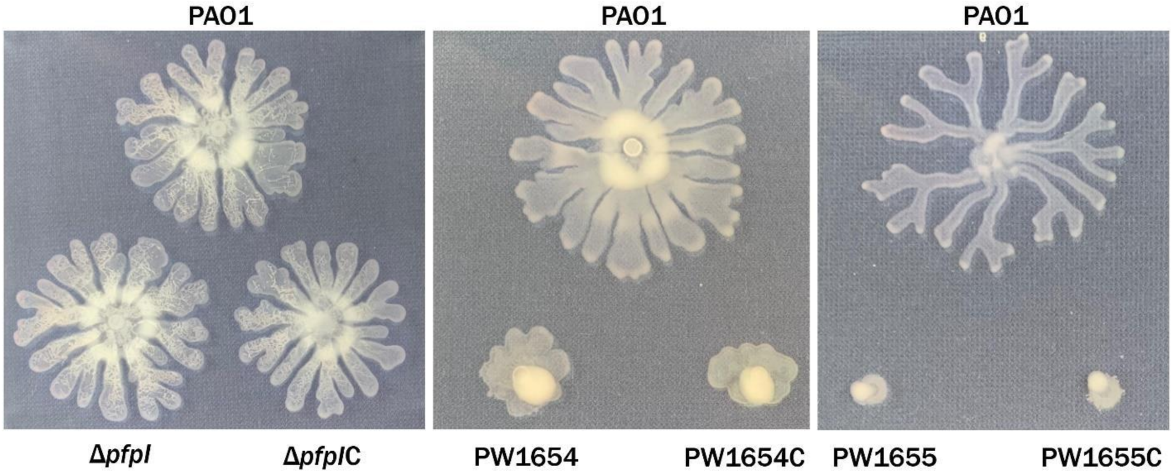

Figure S6. **The surface swarming phenotype associated with PAO1, the Δ*pfpI* mutant (this study) and the UWGC Tn mutants, PW1654 and PW1655**. Swarm assays were carried out as described in the *Materials and Methods*. Photographs of the swarms were made 18 h after inoculation. Δ*pfpI*C, PW1654C and PW1655C indicate strains containing pUCP20(*pfpI*). The swarm plates did not contain selection (carbenicillin) for this plasmid, but the overnight cultures used to inoculate the plates did. Note that PW1654 and PW1655 show defective swarming, and that this phenotype is not complemented by expression of *pfpI* from pUCP20 *in trans*.

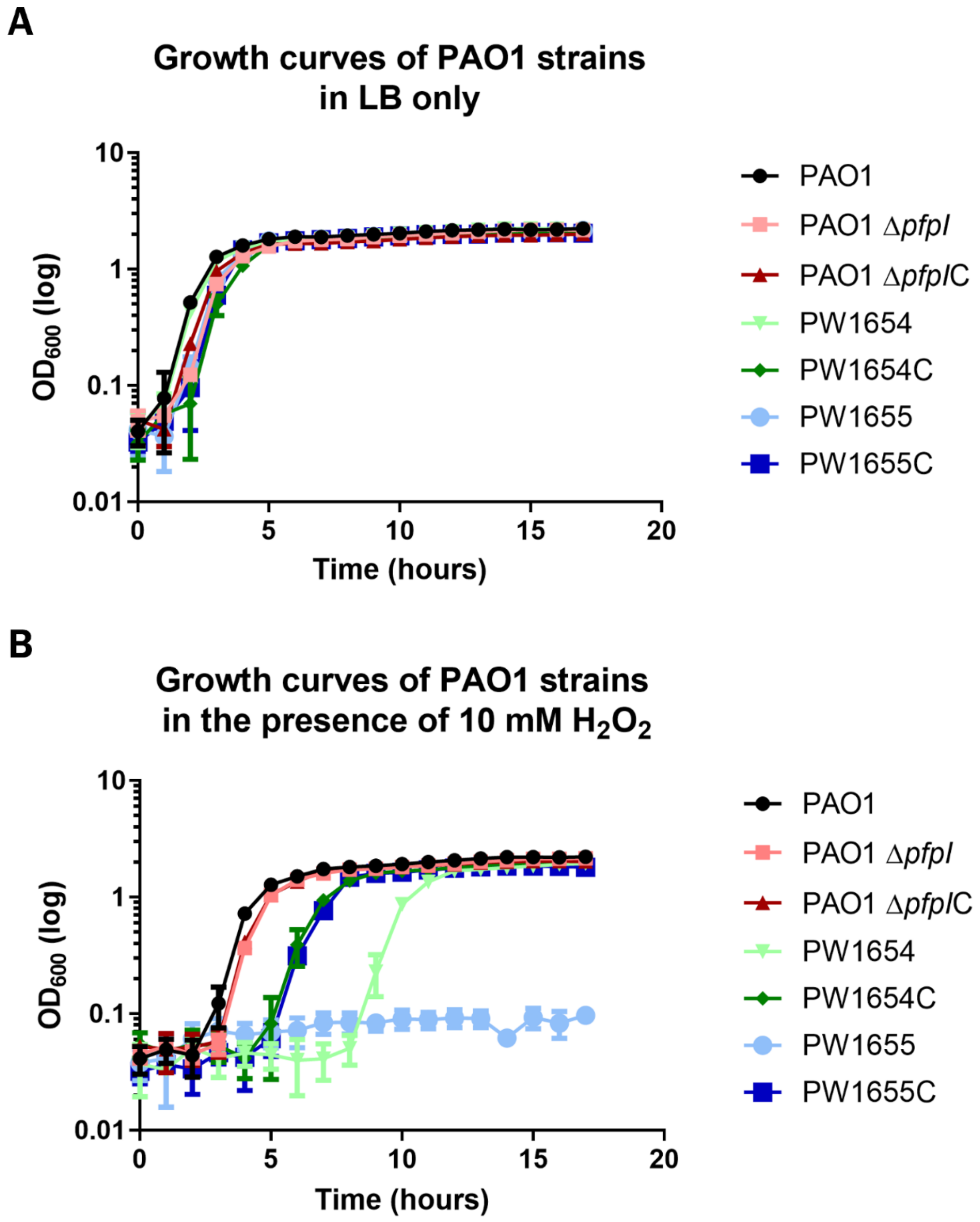

Figure S7. **H_2O_2 sensitivity of PAO1, the Δ*pfpI* mutant (this study) and the UWGC Tn mutants, PW1654 and PW1655**. The figure shows growth curves of the indicated strains grown in (**A**) LB alone, or (**B**) in LB supplemented with 10 mM H_2O_2. N = 3 independent measurements were made for each data point, and the error bars indicate standard deviations. Δ*pfpI*C, PW1654C, and PW1655C indicate strains containing pUCP20(*pfpI*). The cultures did not contain selection (carbenicillin) for this plasmid, but the overnight cultures used for inoculation did. Note the difference in behavior between the progenitor (PAO1), the Δ*pfpI* mutant, and the two Tn mutants (PW1654 and PW1655).

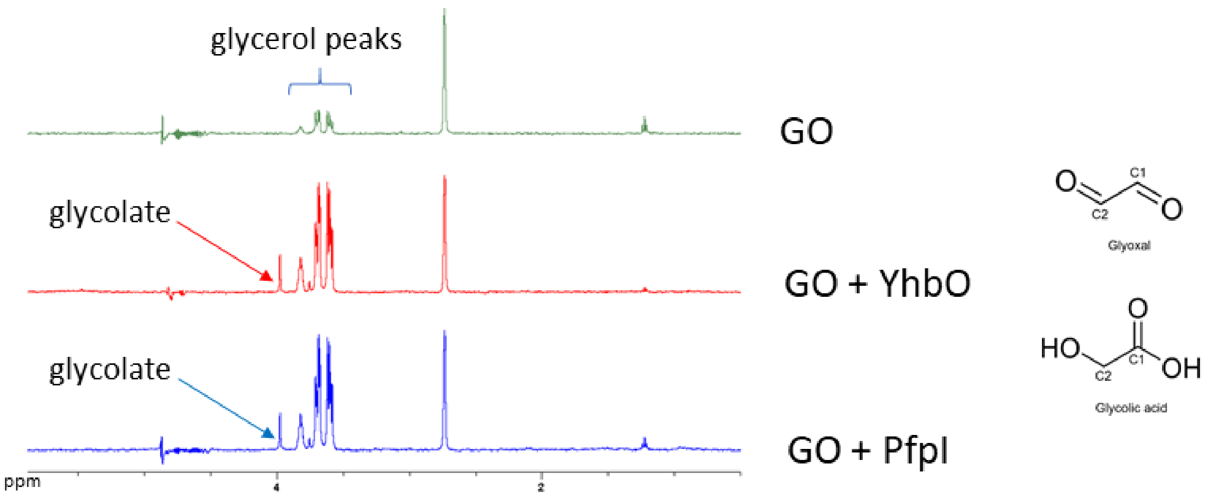

Figure S8. PfpI catalyzes the conversion of glyoxal to glycolate. Purified PfpI (20 µg) or YhbO (20 µg), as indicated, were mixed with glyoxal (GO, 1 mM) and incubated overnight at 4˚C prior to 1D NMR analysis of the reaction end product(s). The green spectrum shows the [^{1}H] NMR spectrum of GO incubated overnight in the absence of PfpI or YhbO. Note the triplet at 1.25 ppm; this is 1,2-ethandiol; a common contaminant of glyoxal solutions. The main glyoxal peak is masked by the water suppression pulse sequence (WATERGATE W5) at approximately 5 ppm; ChemDraw Ultra predicts a doublet in this location. Two sets of peaks can be identified at 3.7 - 3.8 ppm, indicating the presence of glycerol (likely derived from the enzyme preparation). The red spectrum was recorded after overnight incubation with the known glyoxalase, YhbO, whereas the blue spectrum was recorded after overnight incubation with PfpI. The glycolate peaks (which appear only after overnight incubation with the enzymes) are arrowed. These data strongly suggest that PfpI quantitatively converts GO to glycolic acid.

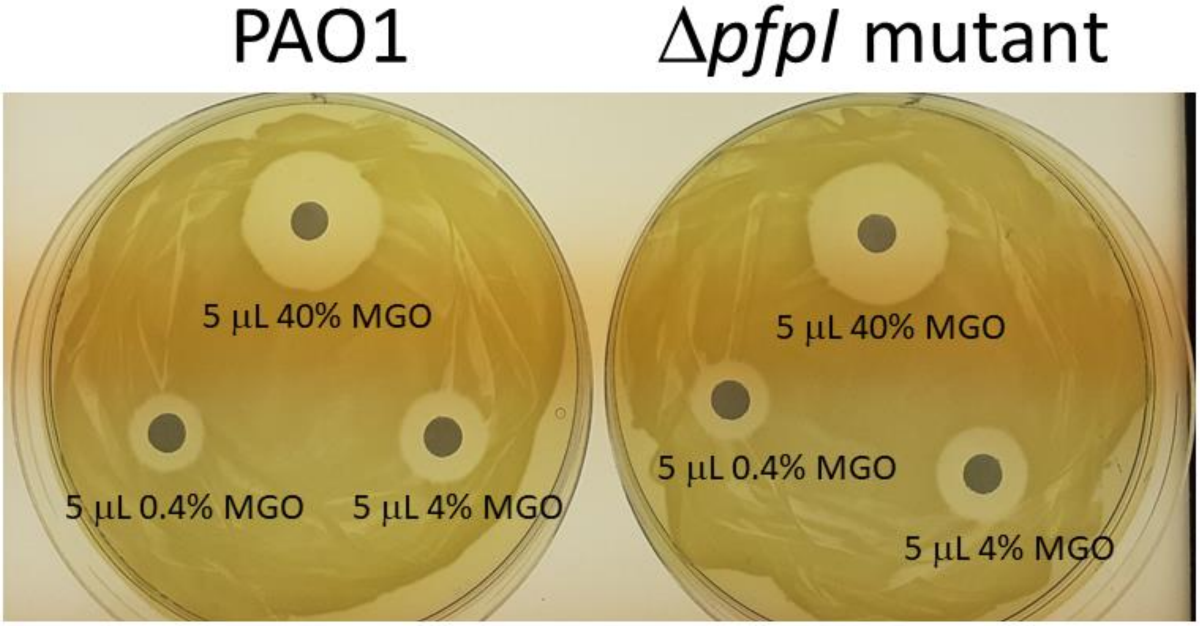

Figure S9. The Δ*pfpI* mutant displays the same sensitivity to exogenous methylglyoxal (MGO) as the wild type during growth on LB-agar. Overnight LB-grown cultures of the wild type and Δ*pfpI* mutant were spread onto LB-agar (1.6% w/v) plates. At the indicated locations, sterile chads of Whatman paper were placed on the surface of the plates using sterile tweezers. Aliquots (5 μL) of MGO (at the indicated concentrations, diluted in sterile water) were then placed onto the chads, and the plates were left overnight at 37°C in a humidified incubator. The plates were photographed 18 h later.
